# Hepatic supersulfides attenuate acetaminophen-induced liver injury via enhanced detoxification and anti-inflammatory mechanisms

**DOI:** 10.1016/j.redox.2026.104140

**Published:** 2026-03-28

**Authors:** Chunyu Guo, Touya Toyomoto, Hiroyasu Tsutsuki, Yukio Fujiwara, Yoshihiro Komohara, Tianli Zhang, Hina Honda, Stephen Lindahl, Takuro Niidome, Jun Fang, Ming Xian, Tomohiro Sawa

**Affiliations:** a Department of Microbiology, Graduate School of Medical Sciences, Kumamoto University, Honjo 1-1-1, Chuo-ku, Kumamoto, Japan; b Transkingdom Signaling Research Unit, Division of Host Defense Mechanism, Tokai University School of Medicine, Japan; c Department of Cell Pathology, Graduate School of Medical Sciences, Kumamoto University, Honjo 1-1-1, Chuo-ku, Kumamoto, Japan; d Comprehensive Center for Infectious Disease Control, Akita University Graduate School of Medicine, Hondo 1-1-1, Akita, Japan; e Key Research Laboratory at Akita University, Hondo 1-1-1, Akita, Japan; f Department of Chemistry, Brown University, Providence, RI, 02912, USA; g Faculty of Advanced Science and Technology, Kumamoto University, Kumamoto, Japan; h Faculty of Pharmaceutical Science, Sojo University, Ikeda 4-22-1, Kumamoto, Japan

**Keywords:** Acetaminophen, Supersulfides, Macrophage polarization, NAPQI, Acute liver injury

## Abstract

Acetaminophen (APAP) is a widely used antipyretic and analgesic agent; however, overdose can lead to hepatotoxicity and, in severe cases, acute liver failure. Development of therapeutics that mitigate APAP-induced liver injury is essential to prevent progression to hepatic failure. Upon overdose, APAP is metabolized in the liver to the highly reactive electrophile *N*-acetyl-*p*-benzoquinone imine (NAPQI), which induces hepatocellular damage. Glutathione (GSH), a key intracellular nucleophile, exists partially in a modified form as glutathione hydropersulfide (GSSH), which exhibits enhanced nucleophilicity and functions as a supersulfide. While detoxification of NAPQI via GSH conjugation is well established, the role of GSSH in NAPQI detoxification has remained unknown. In this study, we investigated the protective role of hepatic supersulfides against APAP-induced liver injury using a murine model. Utilizing a newly developed tandem mass spectrometry technique, we demonstrated that supersulfides form conjugates with NAPQI, which are subsequently excreted in the urine. Moreover, administration of supersulfide donors, such as *N*-acetylcysteine (NAC) tetrasulfide and thioglucose tetrasulfide, elevated hepatic supersulfide levels and significantly attenuated APAP-induced liver injury. Notably, the protective effects of these donors surpassed those of conventional NAC treatment. Our findings suggest that the hepatoprotective effects of supersulfide donors involve not only enhanced detoxification of NAPQI, thereby reducing hepatocellular damage, but also suppression of inflammation. These results highlight the therapeutic potential of targeting hepatic supersulfides in the treatment of APAP overdose.

## 1 Introduction

Acetaminophen (APAP) is a widely used antipyretic and analgesic drug in clinic [1]. Although the therapeutic dose of APAP is relatively safe, excessive doses can cause acute liver injury and even liver failure [2]. Currently, APAP overdose is the leading cause of drug-induced liver injury and even liver failure in the United States, Britain and other western countries [3,4]. However, therapeutic options for managing APAP-induced liver injury remain limited [1,2]. Previous studies have primarily focused on liver regeneration and their signaling mechanisms, which are involved in restoring lost cell mass through hepatocyte proliferation and coordinated angiogenesis [5]. However, cell cycle progression cannot begin until 12-24 h after injury, with cell proliferation typically starting at 24-30 h and peaking at 30-36 h post-injury [5]. Although the mechanisms of liver regeneration have been studied in detail, how to preserve liver function and protect hepatocytes from immediate damage remains unclear. Therefore, developing effective therapeutic strategies to protect liver function and prevent hepatocyte death is crucial.

Inflammation is an another critical event in APAP-induced liver injury, with macrophage polarization playing a pivotal role [6,7]. Macrophages are typically recruited from monocytes upon activation by pro-inflammatory cytokines, which are initially polarized into pro-inflammatory (M1) macrophages to initiate the inflammatory response, followed by polarization into anti-inflammatory (M2) macrophages that promote tissue regeneration and repair [[8], [9], [10]]. Impeding M1 polarization and promoting M2 polarization in macrophages represents a promising therapeutic strategy for the treatment of APAP-induced liver injury [11,12].

APAP metabolism in the liver is almost completely mediated by glucuronidation and sulfation, producing non-toxic metabolites that are then excreted by the kidneys in urine [2,4]. Excess APAP that cannot be detoxified through glucuronidation or sulfation is metabolized in hepatocytes by cytochrome P450 (CYP) system, specifically via CYP2E1 and CYP1A2 metabolism [13], into the highly electrophilic metabolite *N*-acetyl-p-benzoquinone imine (NAPQI). Owing to its strong electrophilicity, NAPQI covalently binds to the nucleophilic thiol group of glutathione (GSH), resulting in the formation of GSH-APAP adduct (G-*S*-APAP) [2,[14], [15], [16]]. This conjugate is subsequently metabolized to a cysteine-APAP adduct (Cys-*S*-APAP), followed by acetylation to form an *N*-acetylcysteine (NAC)-APAP adduct (NAC-*S*-APAP), both of which are ultimately excreted in the urine [15,16]. In cases of APAP overdose, intracellular GSH is rapidly depleted due to excessive reaction with NAPQI, thereby impairing the formation of detoxifying G-*S*-APAP conjugates. Consequently, unneutralized NAQPI reacts with protein thiol groups, leading to hepatocellular damage characterized by liver necrosis and subsequent hepatic inflammation [2,4,14,17].

Recent advances in redox biology have demonstrated that GSH can undergo further sulfur modification to form glutathione hydropersulfide (GSSH), which is endogenously present in biological tissues [18,19]. GSSH and related cysteine (CysSH)-based persulfides such as cysteine hydropersulfide (CysSSH) exhibit markedly increased nucleophilicity compared to GSH and cysteine, thereby enhancing their reactivities toward electrophilic substances [[20], [21], [22], [23], [24]]. Owing to their elevated chemical reactivities, persulfides, including GSSH, display unique biological activities that are not observed in their parent thiols and are collectively referred to as “Supersulfides” [[20], [21], [22], [23], [24]].

It is thus hypothesized that supersulfides contribute to detoxification processes through direct reaction with electrophilic NAPQI. However, the in vivo reactivity between NAPQI and supersulfides has not been elucidated, and their potential involvement in the pathogenesis of APAP-induced hepatotoxicity remains unclear. In the present study, we investigated the endogenous formation of GSSH-APAP conjugates (G-*S*_*2*_-APAP) in response to APAP overdose. To this end, we developed a highly sensitive and specific mass spectrometry-based quantification method for G-*S*_*2*_-APAP and applied it to analyze persulfides-APAP adducts in liver, blood, and urine obtained from mice.

To further evaluate the protective roles of supersulfides against APAP-induced hepatotoxicity, we assessed the therapeutic potentials of supersulfide donors. We previously reported that NAC tetrasulfide (NAC-S2) as well as thioglucose tetrasulfide (TGS4) serve as effective donors of supersulfides [[25], [26], [27], [28], [29]] (Supplementary Fig. S1). Oxidized NAC (oxNAC), that lacks sulfur donating capability, is used as a negative control for NAC-S2. Given our prior findings that supersulfide donors exhibit anti-inflammatory properties, we also examined the impact of those donors on macrophage polarization toward pro-inflammatory phenotype in APAP-overdose mice.

## 2 Methods

### 2.1 Regents

The supersulfide donors, NAC-S2 and TGS4, along with the control compound oxNAC, were synthesized following the previous studies [25,26]. APAP Intravenous Injection (M22055) was obtained from Terumo Corporation (Tokyo, Japan). APAP (A5000-100G) and protease from *Streptomyces griseus* (53702-25KU) were obtained from Sigma-Aldrich (St. Louis, MO, USA). NAC (616-91-1), NAPQI (50700-49-7), Protein Assay BCA Kit (297-73101) and ALT/AST CⅡ Test Kit (431-30901) were purchased from FUJIFILM Wako Pure Chemical Corporation (Osaka, Japan). Mouse IL-1β/IL-1F2 Quantikine ELISA Kit (MLB00C), Mouse IL-6 Quantikine ELISA Kit (M6000B-1), and Mouse IL-10 Quantikine ELISA Kit (M1000B) were obtained from R&D Systems (Minneapolis, MB, USA). Primary antibodies of inducible nitric oxide synthase (iNOS, sc-651) and arginase-1 (Arg-1, sc-271430) were obtained from Santa Cruz Biotechnology (Santa Cruz, CA, USA); and primary antibody of β-actin (010-27841) was purchased from FUJIFILM Wako Pure Chemical Corporation (Osaka, Japan). Primary antibodies of Glutamate–cysteine ligase catalytic subunit (GCLC, ab190685) were obtained from Abcam (Cambridge, UK). Secondary anti-mouse IgG HRP-linked antibodies (7076) was obtained from Cell Signaling Technology (Danvers, MA, USA).

### 2.2 Animals and experiment design

Adult male ICR mice (8 weeks, 36-38 g) were purchased from Japan SLC Inc. (Shizuoka, Japan). All mouse experiments were approved form the Kumamoto University Ethics Review Committee for Animal Experimentation (approval number: A2020-065). The mice were allowed to acclimate for one week before the experiment, during which they had free access to food and water. After acclimation, mice were randomly assigned to experimental groups and fasted for 12 h prior to APAP administration and until euthanasia. Mice received a single intraperitoneal (i.p.) injection of APAP (330 mg/kg) to induce acute liver injury and were sacrificed at designated time points depending on the experiment.

(1) Efficacy evaluation of NAC-S2 and TGS4: In the NAC-S2 study, five groups were established (control, APAP, and three NAC-S2 treatment groups; n = 8 per group, n = 5 for control). NAC-S2 was administered subcutaneously (s.c.) at 0.5 and 2 h post-APAP at doses of 3/6, 10/20, or 30/60 mg/kg, with 10/20 mg/kg identified as the optimal dose. To compare efficacy, an additional five groups (control, APAP, NAC-S2, oxNAC, NAC) received equimolar doses of NAC-S2 (10/20 mg/kg), oxNAC (8.4/16.8 mg/kg), or NAC (8.4/16.8 mg/kg) at the same time points. NAC-S2 alone was also administered (10/20 and 30/60 mg/kg) to assess safety. In the TGS4 study, four groups (control, APAP, and two TGS4 treatment groups; n = 8 per group, n = 5 for control) received TGS4 subcutaneously at 0.5 and 2 h post-APAP at doses of 3.9/7.8 and 11.7/23.4 mg/kg, corresponding to the molar equivalents of NAC-S2 (3/6 and 10/20 mg/kg, respectively).
(2) GSH and cysteine metabolomic analysis: Mice were divided into three groups (control, APAP, and NAC-S2; n = 7 per group, n = 5 for control). NAC-S2 was administered s.c. at 10/20 mg/kg at 0.5 and 2 h post-APAP, respectively. Mice were euthanized 4 h after APAP administration. In addition, mice were divided into two groups (control and NAC-S2). NAC-S2 was administered s.c. at 10/20 mg/kg, with a 1.5-h interval between the two administrations. Mice were euthanized either 2 h or 15 min after the second administration.
(3) APAP metabolomic analysis: Mice were divided into two groups (APAP and NAC-S2; n = 3 per group). NAC-S2 (10 or 20 mg/kg) was administered s.c. 0.5 h after APAP injection. Mice were sacrificed 1 h post-APAP. Additionally, liver tissues from the GSH and cysteine metabolomics experiment were also used for APAP metabolite analysis.

### 2.3 Cell culture and treatment

HepG2 cells were cultured in EMEM medium (Osaka, Japan) supplemented with 10% heat-inactivated fetal bovine serum and 1% penicillin–streptomycin in a humidified incubator at 37 °C with 5% CO_2_. Cells were seeded at a density of 1 × 10^6^ cells per well in 6-well-plate. After overnight incubation, cells were treated with 150 μM oxNAC or 150 μM NAC-S2 for 30 min, followed by medium replacement and treatment with 5 mM APAP. Thirty minutes later, both the supernatants and cells were collected for APAP metabolite analysis.

### 2.4 Synthesis and purification of APAP adducts

Five μl of 100 mM cysteine, GSH, and NAC were reacted with 5 μl of Na_2_S_2_ (at concentrations of 100 mM, 50 mM, and 50 mM, respectively) in 35 μl of 50 mM sodium phosphate buffer (pH 7.4) at 37 °C for 5 min. The mixtures were then added with 5 μl of 50 mM NAPQI and incubated at 37 °C for 30 min. Subsequently, the mixtures were diluted 10-fold in 0.1% formic acid for APAP adducts purification by high performance liquid chromatography (HPLC) (Agilent Technologies, Santa Clara, CA, USA). The mixtures (150 μl) were injected into a YMC-Pack ODS-AQ (4.6 × 250 mm; YMC Co., Ltd., Kyoto, Japan). The mobile phases consisted of 0.1% formic acid and acetonitrile, with an acetonitrile gradient changed from 1% to 80% (for cysteine mixture), 90% (for GSH mixture), 90% (for NAC mixture) in 16 min with the flow rate of 0.8 ml/min. The APAP adducts were detected at 246 nm and determined by liquid chromatography-tandem mass spectrometry (LC-MS/MS) (Agilent Technologies, Santa Clara, CA, USA). The corresponding peak of APAP adducts were collected and then subjected to lyophilization. The APAP adducts were dissolved in 50 μl of H_2_O, and their concentrations were determined based on a 100 μM APAP standard by LC-MS/MS. The concentration of the APAP adducts were then adjusted to 5 μM.

### 2.5 Metabolomic analysis of APAP adducts and sulfur-containing molecules

Metabolomic analysis of analytes, including APAP adducts, CysSH, GSH, and their related supersulfides, was performed on samples collected as soon as possible, using LC-MS/MS. Specifically, liver tissues (10-20 mg) were homogenized in 5 mM HPE-IAM, and bladder urine (5 μl) and blood (10 μl) were mixed with 5 mM HPE-IAM [25]. The samples were incubated at 37 °C for 30 min, and then centrifugated at 13000 rpm for 10 min. The supernatants were diluted 10-fold in 0.1% formic acid for multiple reaction monitoring (MRM) analysis. The amounts of all analytes were determined by comparison to the corresponding standards. Among these analytes, CysSH, GSH, and their related supersulfides were indirectly quantified via stabilized HPE-IAM adducts. Simultaneously, the centrifuged precipitates of liver tissue were lysed with 1% sodium dodecyl sulfate (SDS) in PBS to determine the protein concentration using a BCA kit. The data are presented as pmol/mg protein for liver tissue, and nM for bladder urine and blood.

### 2.6 Metabolomic conditions

The LC-MS/MS conditions were as follows: column, YMC-Triart C18 Plus column (2.1 × 50 mm) (YMC Co. Ltd., Kyoto, Japan); column temperature, 45 °C; injection volume, 1 μL; mobile phases: A, 0.1% formic acid, and B, acetonitrile; gradient (B concentration), 0 min – 1%, 10 min – 80%, 10.1 min – 1%, 15 min – 1%; and flow rate, 0.2 mL/min. The general conditions for electrospray ionization-mass spectrometry were nebulizer gas, nitrogen, delivered at 50 psi; nebulizer gas temperature, 250 °C; capillary voltage, 3500 V; collision gas, and G1 grade, nitrogen (Taiyo Nippon Sanso Corporation, Tokyo, Japan). Supplementary Table 1 provides the MRM parameters for all analytes used in this study.

### 2.7 Analysis of protein-bound Cys-S-APAP, 3-nitrotyrosine (3-NO_2_-Tyr) and tyrosine (Tyr)

Protein-derived Cys-*S*-APAP, 3-NO_2_-Tyr and Tyr were quantified using LC-MS/MS, based on previously described methods with some modifications [30]. Briefly, liver tissues (50 mg) were homogenized in 500 μl of 10 mM sodium acetate, sonicated for 1 min, and centrifuged at 13,000 rpm for 10 min. Supernatants and protease (8 U/mL) were subjected to ultrafiltration using Centrifugal Filters with a 10 kDa molecular weight cut-off, repeated three times to remove free Cys-*S*-APAP and other small molecules that might interfere with the assay. The filtrated samples were then incubated with equal volume of filtrated protease at 50 °C for 18 h. After protease digestion, the samples were again filtered, and the ultrafiltrates were collected for MRM analysis. Similarly, the centrifuged precipitates of liver tissue were lysed in 0.1 % SDS buffer, and total protein concentration was determined using a BCA kit. The data are presented as pmol/mg protein.

### 2.8 Quantification of serum alanine aminotransferase (ALT) and aspartate aminotransferase (AST)

Blood samples were collected from the tail vein at designated time points and from the inferior vena cava at the time of euthanasia. Samples were left at room temperature for 2 h, then centrifuged at 3500 g for 15 min at 4 °C to obtain serum. Serum ALT and AST levels were measured using the ALT/AST CⅡ Test Kit according to the manufacturer's instructions.

### 2.9 Histopathological examination

Approximately 5 mm × 5 mm liver tissue was collected from the center of the left lateral lobe of the mouse liver, fixed in 4% paraformaldehyde for 24 h, and then embedded in paraffin. The paraffin-embedded tissue was sectioned into 3 μm thick slices and stained with hematoxylin and eosin (H&E). The liver necrosis area (%) was analyzed and quantified from the H&E staining images using HALO image analysis software (Indica Labs, NM, USA).

### 2.10 Immunohistochemistry

Mouse liver tissue specimens were cut into 3-μm sections. The sections were deparaffinized and hydrophilized, followed by antigen retrieval, endogenous peroxidase removal, and protein-blocking. The primary antibodies used were anti-F4/80 (ab111101, Abcam, Cambridge, UK), and anti-Myeloperoxidase (MPO) (A0398; DAKO, Agilent Technologies, Santa Clara, CA, USA). Samples were then incubated with peroxidase-labeled goat anti-rabbit secondary antibodies (#413341; Nichirei Biosciences). Immunoreactions were visualized using the Diaminobenzidine Substrate Kit (#425011; Nichirei Biosciences). The images were captured using a NanoZoomer digital slide scanner (Hamamatsu Photonics, Hamamatsu, Japan). The acquired images were analyzed using the HALO image analysis software (Indica Labs, Albuquerque, NM, USA) to assess distribution of F4/80^+^ and MPO^+^ cells within the tissue microenvironment.

### 2.11 Quantification of cytokines

Liver tissues (90-100 mg) were homogenized and lysed in RIPA buffer [150 mM NaCl, 50 mM Tris-HCl (pH 7.4), 1% TritonX-100, 1% sodium deoxycholate, 0.1% SDS] containing 1% protease inhibitor and 1% phosphatase inhibitor. The liver lysates were centrifuged at 12,000 g at 4 °C for 15 min, and the supernatants were collected. And the supernatants were quantified by Protein Assay BCA Kit. Then the lysis supernatants were used to measure the IL-1β, IL-6 and IL-10 levels using the Mouse IL-1β/IL-1F2 ELISA Kit, the Mouse IL-6 ELISA Kit, and the Mouse IL-10 ELISA Kit according to the manufacturer's instructions. Absorbance at 490 nm was detected by iMark Microplate Absorbance Reader (Bio-Rad Laboratories, Hercules, CA, USA). The cytokine levels were calculated by standard curve and the data are presented as pg/mg protein.

### 2.12 Western blotting

The lysates prepared as described above were quantified for protein concentration and then combined with SDS loading buffer (62.5 mM Tris-HCl pH 6.8, 6% glycerol, 2% SDS, 0.005% bromophenol blue, and 2.5% 2-mercaptoethanol). The samples were heated at 98 °C for 5 min to achieve denaturation and reduction. Equal amounts of protein were then loaded onto 10% polyacrylamide gels, and proteins were separated by means of SDS–polyacrylamide gel electrophoresis (SDS-PAGE). Subsequently, separated proteins were transferred to polyvinylidene difluoride (PVDF) membranes (Merck Millipore, Darmstadt, Germany) at 100 V for 1 h, which were blocked with 5% of non-fat milk in TBS-T (20 mM Tris pH 7.6, 137 mM NaCl, and 0.1% Tween 20) at room temperature. One hour later, membranes were incubated with primary antibodies specific for iNOS, Arg-1, GCLC and β-actin, respectively. After an overnight incubation at 4 °C, membranes were washed three times with TBS-T, followed by incubation with HRP-linked anti-mouse IgG secondary antibodies at room temperature for 1 h. After the membranes were washed again with TBS-T, the target protein bands on the membranes were detected with Immobilon Western Chemiluminescent HRP Substrate (Merck Millipore) and visualized using a luminescent image analyzer ChemiDoc XRS system (Bio-Rad Laboratories). The band intensity was analyzed by ImageJ software and normalized by β-actin.

### 2.13 Statistical analysis

All data in this study are presented as mean ± standard deviation (SD). Statistical analysis was performed using GraphPad Prism (version 8.0; GraphPad Software, La Jolla, CA, USA). Student's *t*-tests were performed to assess differences between two groups. For comparisons among multiple groups, one-way analysis of variance (ANOVA) was conducted, followed by Tukey's post-hoc multiple comparison test. Statistical significance was set at a *P* value of <0.05.

## 3 Results

### 3.1 Preparations of authentic supersulfide-APAP conjugates

**Fig. 1:**
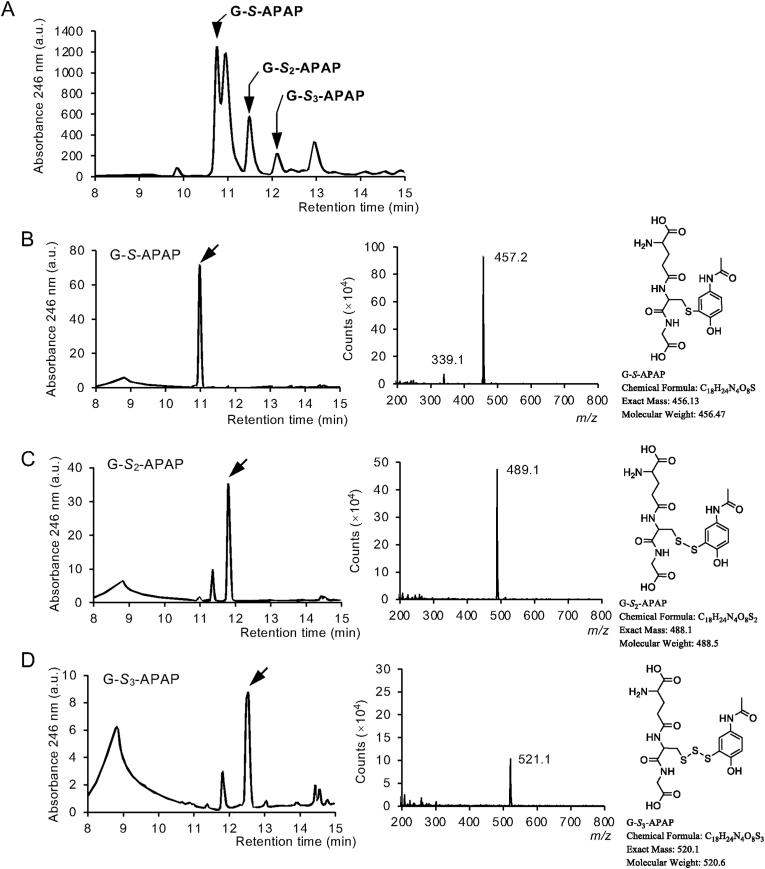
**Characterization of glutathione supersulfide-APAP adducts.** (A) Reverse-phase HPLC separation for reaction mixture of GSH, Na_2_S_2_, and NAPQI. Peaks correspond to G-*S*_*n*_-APAP adducts (n, 1∼3) indicated by arrows were collected and subjected for HPLC and mass spectrometry analyses. Characterization of (B) G-*S*-APAP, (C) G-*S*_2_-APAP, and (D) G-*S*_3_-APAP. Left panels show HPLC chromatograms, middle panels show mass chromatograms, and right panels show chemical structures.

Because authentic supersulfide-APAP conjugates, that were necessary for mass spectrometry-based analysis, were not commercially available, we first synthesized and purified the standards of APAP conjugates (Supplementary Fig. S2). For example, thiol moiety of GSH could be converted to persulfide and trisulfide in the presence of Na_2_S_2_ under ambient conditions. To the solution of GSH and Na_2_S_2_ was added NAPQI, resulting in the formation of APAP adducts of GSH and its supersulfides. As shown in Fig. 1A, APAP adducts thus formed could be separated by means of reverse-phase HPLC. Fractions containing each APAP adduct were collected and subjected for mass spectrometry analyses and HPLC analyses (Fig. 1B–D). Similar protocols were employed to prepare cysteine supersulfide- and NAC supersulfide-APAP adducts (Supplementary Figs. S3 and S4).

### 3.2 LC-MS/MS-based quantification of APAP conjugates

By using authentic supersulfide-APAP conjugates just prepared above, we developed sensitive quantification method for APAP conjugates by means of LC-MS/MS. We employed MRM method for quantification of APAP conjugates. As shown in Fig. S5, all APAP conjugates analyzed could be determined by this method in a range of 5 fmol to 5 nmol with good linearity. Detection limits of APAP conjugates were approximately 5 fmol/injection for each conjugate (signal-to-noise ratio was larger than 15).

### 3.3 Endogenous formations of supersulfide-APAP metabolites

**Fig. 2:**
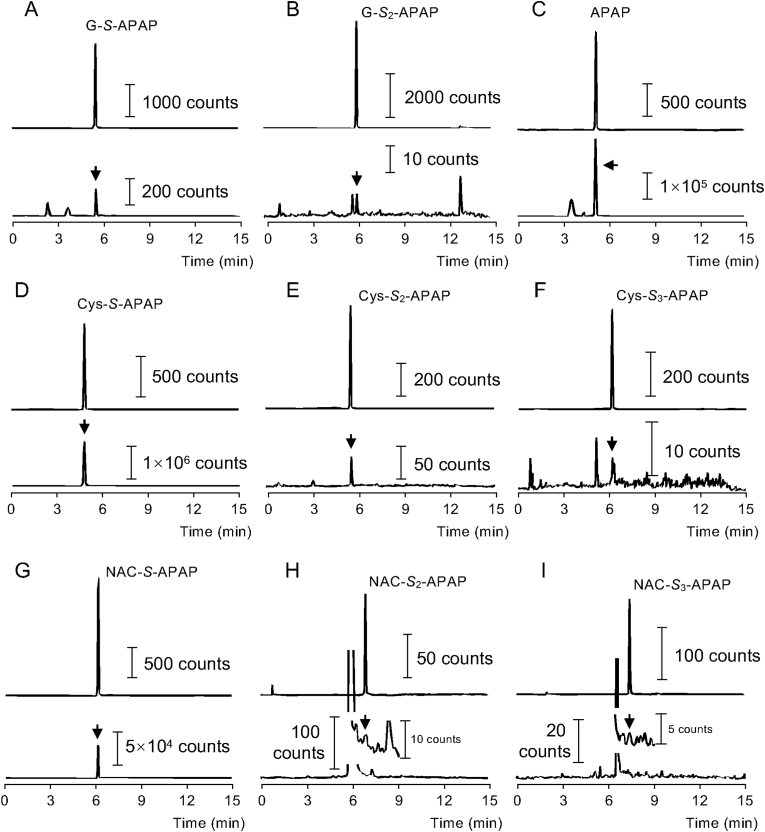
**Identification of supersulfide-APAP adducts in urine from mice administered APAP.** Mice were administered APAP (330 mg/kg, intraperitoneally), and urine was collected 1 h post-dosing. Urinary APAP conjugates were quantitatively analyzed by LC-MS/MS (A-I). In each panel, the upper trace shows the chromatogram of the authentic standard, and the lower trace shows the corresponding urinary sample. Arrowheads indicate peaks correspond to the authentic standards.

Following intraperitoneal administration of APAP at a dose of 330 mg/kg in mice, urine samples were collected from the bladder and analyzed for APAP conjugates by means of LC-MS/MS. As shown in Fig. 2G–S-APAP, Cys-*S*-APAP, and NAC-*S*-APAP were clearly detected in urine samples. In addition, unchanged APAP was also excreted in the urine. Notably, distinct peaks corresponding to G-*S*_*2*_-APAP, CysSSH-APAP adduct (Cys-*S*_*2*_-APAP), and cysteine polysulfide APAP adduct (Cys-*S*_*3*_-APAP) were observed. These findings indicate that endogenous supersulfides react with NAPQI, and that the resulting metabolites are excreted in the urine. To the best of our knowledge, this is the first report demonstrating the detection of persulfide-APAP conjugates upon APAP overdose model in vivo.

### 3.4 Enhanced detoxification of NAPQI via supersulfide conjugation by supersulfide donor treatment

**Fig. 3:**
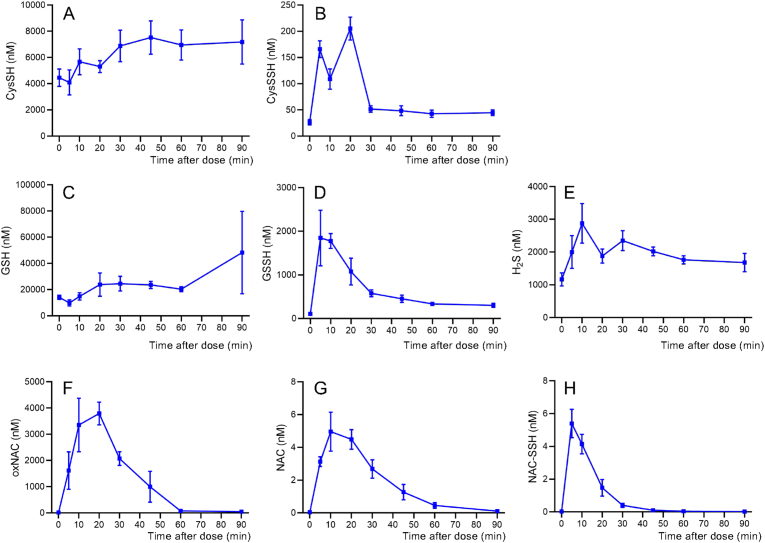
**Blood concentrations of sulfur metabolites in mice after subcutaneous administration of NAC-S2.** Mice were administered NAC-S2 (1.4 mg/kg) subcutaneously. At designated time points, blood samples (10 μL) were collected from tail vain and subjected to tandem mass spectrometric quantification of sulfur metabolites. Metabolites analyzed were (A) cysteine, (B) CysSSH, (C) GSH, (D) GSSH, (E) H_2_S, (F) oxNAC, (G) NAC, and (H) NAC-SSH. Data are expressed as mean ± SD (n = 3).

As mentioned above, persulfides exhibit greater nucleophilicity compared to their parent thiols [[20], [21], [22], [23], [24]] and are therefore expected to react more efficiently with electrophilic compounds such as NAPQI. Based on this assumption, increasing the levels of persulfides in the liver may enhance the detoxification of NAPQI and ultimately exert protective effects against APAP overdose-induced liver injury. NAC-S2 is a compound containing a tetrasulfide moiety (Supplementary Fig. S1) and functions as a supersulfide donor capable of transferring sulfur atoms to acceptor thiols [25]. Subcutaneous administration of NAC-S2 to mice resulted in a rapid increase in circulating GSSH and cysteine CysSSH, detectable 5 min after injection. These levels returned to baseline approximately 50 min post-administration (Fig. 3). NAC-S2 itself was not detectable in the circulation at any of the observed time points. Instead, oxNAC, formed by the loss of two sulfur atoms from NAC-S2, was detected in the blood (Fig. 3F). Only trace amounts of reduced NAC and NAC hydropersulfide were observed (Fig. 3G and H). These findings indicate that subcutaneously administered NAC-S2 transfers sulfur atoms to acceptor thiols such as GSH and cysteine, converting itself to oxNAC, while the generated GSSH and CysSSH circulate systemically.

**Fig. 4:**
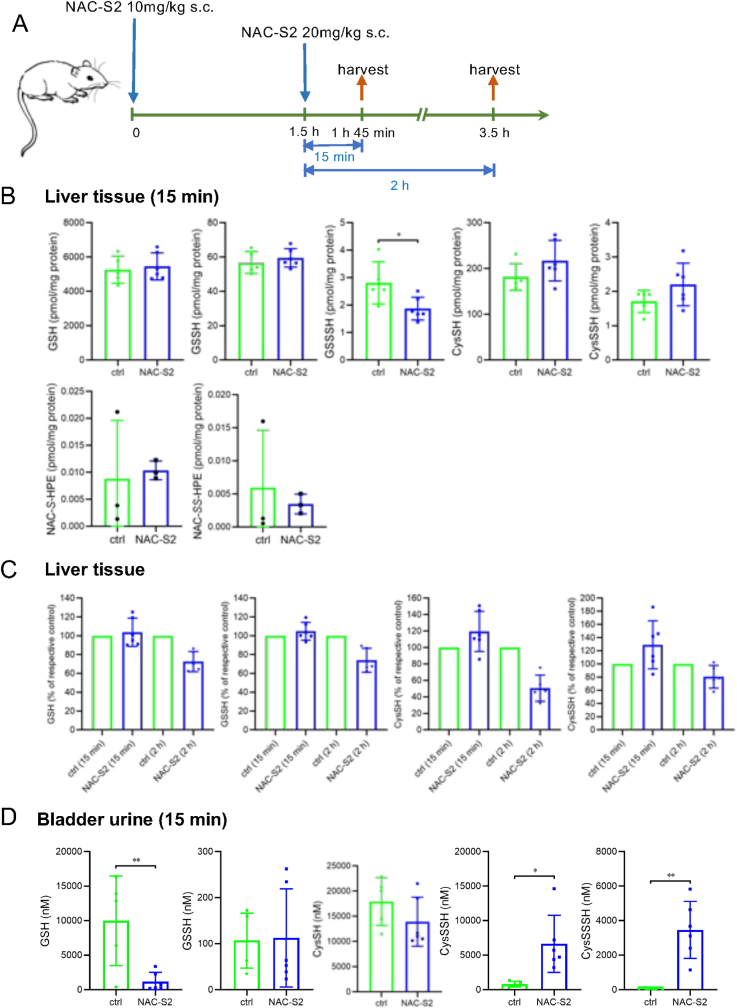
**Effect of NAC-S2 administration on supersulfide levels in the liver and urine of mice.** (A) Experimental protocol. Mice were subcutaneously administered NAC-S2 at 0 h (10 mg/kg) and 1.5 h (20 mg/kg). Liver tissue and bladder urine were collected at 15 min or 2 h after NAC-S2 administration. These samples were subjected to sulfur metabolomic analysis. Levels of GSH, cysteine, and their hydropersulfides in liver tissue (B,C) and urine (D) from mice 15 min after NAC-S2 treatment. Data are expressed as the mean ± SD (n = 5). Statistical analysis was performed using Student's *t*-test. ∗*P* < 0.05, ∗∗*P* < 0.01, ∗∗∗*P* < 0.001. (C) Changes in hepatic levels of GSH, cysteine, and their hydropersulfides following NAC-S2 administration. The levels are expressed relative to the untreated group (set at 100%).

Next, sulfur metabolites in the liver were analyzed following subcutaneous administration of NAC-S2 according to the protocol shown in Fig. 4A. In contrast to the transient increase observed in the circulation, hepatic sulfur metabolites were largely unaffected at both 15 min and 2 h after NAC-S2 administration (Fig. 4B and C). Analysis of urinary metabolites 15 min after NAC-S2 injection revealed a marked increase in CysSSH and cysteine hydrotrisulfide (CysSSSH) in NAC-S2–treated mice (Fig. 4D). These results suggest that although NAC-S2 transiently elevates circulating supersulfide levels in normal mice, transfer of these species to the liver is limited, thereby maintaining hepatic sulfur metabolite homeostasis.

**Fig. 5:**
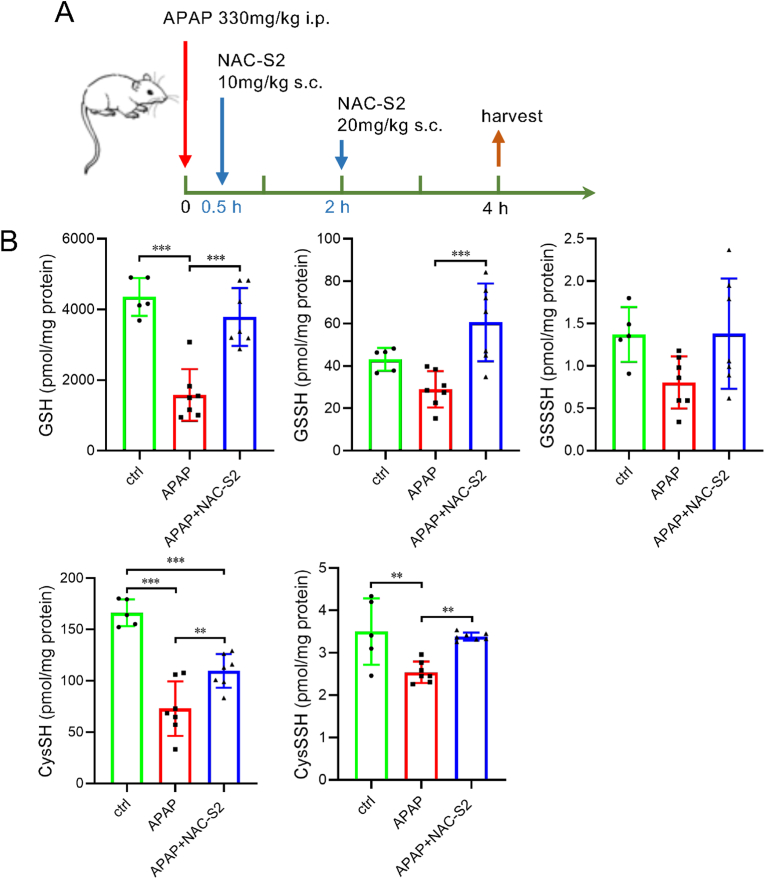
**Effects of supersulfide donor treatment on hepatic supersulfide levels. (A) Experimental protocol.** Mice were treated with NAC-S2 at indicated time after APAP administration, and liver tissue was collected 4 h post-APAP dosing. Liver tissues thus obtained were subjected for sulfur metabolomics. (B) Hepatic levels of GSH, cysteine, and their supersulfides. Statistical analysis was performed using one-way ANOVA followed by Tukey's post hoc multiple comparisons test. Data were expressed by means ± SD (n ≥ 5). ∗*P* < 0.05, ∗∗*P* < 0.01, ∗∗∗*P* < 0.001.

We next investigated how hepatic sulfur metabolites change in mice subjected to APAP overdose and how NAC-S2 administration influences these alterations. Using the protocol illustrated in Fig. 5A, mice were intraperitoneally administered APAP, and liver samples were collected 4 h later. Consistent with previous reports [31], APAP administration resulted in a substantial decrease in hepatic GSH levels (Fig. 5B). Additionally, levels of GSH-derived supersulfides [GSSH and glutathione hydrotrisulfide (GSSSH)] were significantly reduced. Similarly, both cysteine and CysSSH were markedly decreased following APAP administration. These results demonstrate that APAP overdose reduces not only hepatic GSH and cysteine but also their corresponding supersulfide species. In contrast, in the livers of NAC-S2–treated mice, the APAP-induced decreases in GSH, cysteine, and their supersulfides were restored to levels comparable to those in control mice. Unlike normal mice, in which hepatic sulfur metabolites were unaffected by NAC-S2, APAP-injured livers appeared to permit the transfer of circulating GSSH and CysSSH into hepatic tissue. Thus, NAC-S2 administration likely increased hepatic supersulfide levels indirectly by elevating circulating supersulfides, which were subsequently delivered to the damaged liver. We therefore next examined whether NAC-S2 administration enhances urinary excretion of NAPQI adducts, particularly APAP–supersulfide adducts.

**Fig. 6:**
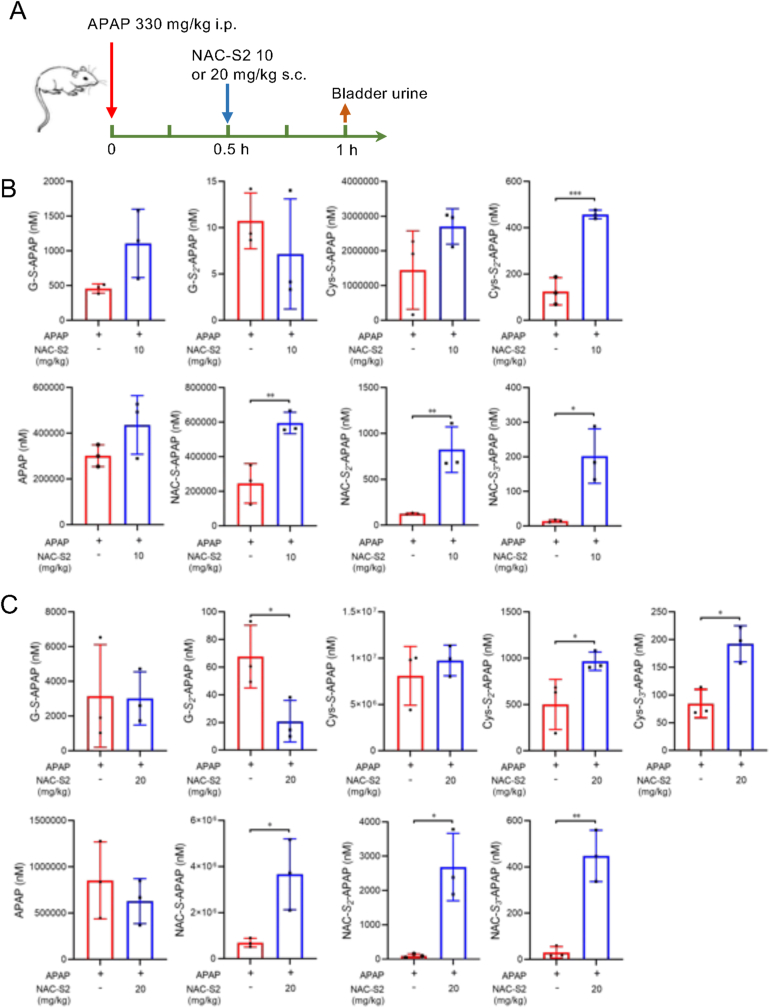
**Enhancement of urinary excretion of supersulfide-APAP conjugates by NAC-S2 treatment.** (A) Experimental protocol. Mice were treated with 10 or 20 mg/mg of NAC-S2 30 min after APAP administration, and bladder urine was collected 1 h post-APAP administration. Levels of APAP adducts in urine were analyzed by LC-MS/MS as shown in (B) for 10 mg/kg NAC-S2 treatment, and (C) for 20 mg/kg NAC-S2 treatment. Data were expressed by means ± SD (n = 3). Statistical analysis was performed using Student's *t*-test. ∗*P* < 0.05, ∗∗*P* < 0.01, ∗∗∗*P* < 0.001.

NAC-S2 was subcutaneously administered 30 min after APAP injection, and urine samples were collected another 30 min later. This experiment employed a single-administration protocol to elucidate the dose-dependent effects of NAC-S2 on the formation of adducts between NAPQI and persulfides generated by APAP overdose. As shown in Fig. 6 and Fig. S6, urine samples from NAC-S2-treated mice contained significantly higher levels of supersulfide-APAP adducts, including Cys-*S*_*2*_-APAP, NAC persulfide APAP adduct (NAC-*S*_*2*_-APAP), and NAC polysulfide APAP adduct (NAC-*S*_*3*_-APAP), compared to vehicle-treated mice. In contrast, these supersulfide–APAP adducts were scarcely detected in the liver or blood, and administration of NAC-S2 did not result in an increase in these adducts (Supplementary Figs. S7 and S8). As described above, blood concentrations of CysSSH and GSSH reached their maximum approximately 5 min after NAC-S2 administration. These data may suggest that NAPQI rapidly formed adducts with these species, which were subsequently excreted into the urine.

The formation of supersulfide–APAP adducts was also observed in human cells when HepG2 cells were treated with APAP. HepG2 cells were exposed to APAP for 30 min, after which APAP adducts present in the culture supernatant and cell lysates were separately analyzed (Supplementary Fig. S9). In addition, groups pretreated with oxNAC or NAC-S2 for 30 min prior to APAP exposure were also examined. As a result, APAP adducts of GSH and cysteine supersulfides were detected in the culture supernatant of APAP-treated cells. These findings indicate that endogenously present GSH and cysteine supersulfides in HepG2 cells react with NAPQI to form adducts. Furthermore, supersulfide–APAP adducts were markedly increased by NAC-S2 pretreatment (Supplementary Fig. S9 B). In contrast, although supersulfide–APAP adducts were detected in cell lysates, their levels were not increased by NAC-S2 treatment (Supplementary Fig. S9 C). This suggests that supersulfide–APAP adducts generated intracellularly are rapidly exported to the extracellular space. NAC-*S*_*2*_- and NAC-*S*_*3*_-APAP adducts were also detected in the culture supernatant following NAC-S2 pretreatment. These NAC-APAP adducts were barely detectable in the absence of NAC-S2 or when oxNAC was added, suggesting that NAC hydropersulfide derived from NAC-S2 directly reacts with NAPQI. Collectively, these results demonstrate that supersulfide–APAP adducts are formed not only in mice but also in human cells.

**Fig. 7:**
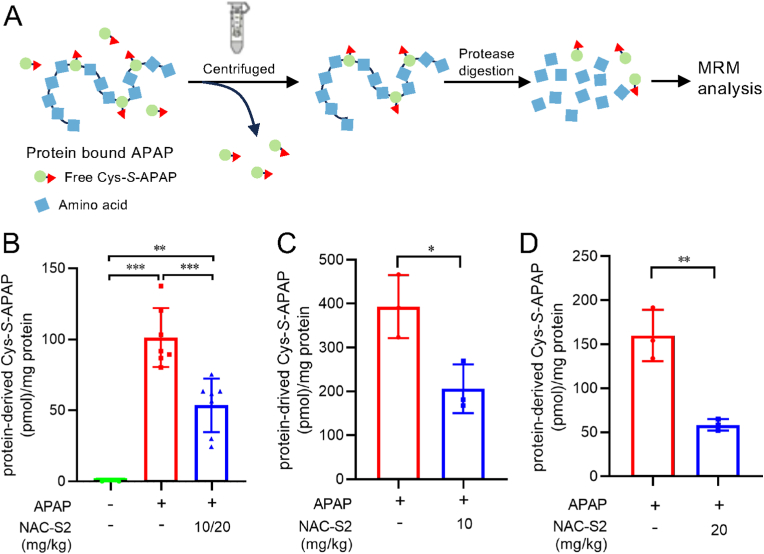
**Reduction of protein-bound APAP adducts in liver tissue by NAC-S2 treatment.** (A) Experimental protocol. Mouse liver homogenates were subjected to ultrafiltration to remove free Cys-*S*-APAP, followed by enzymatic digestion. Cys-*S*-APAP in the digested samples was quantified by mass spectrometry. (B) Protein-bound Cys-*S*-APAP adducts in mouse liver 4 h after APAP administration. Mice were intraperitoneally injected with APAP (330 mg/kg), followed by subcutaneous injection of NAC-S2 (10 mg/kg) at 30 min post-APAP and a second dose of NAC-S2 (20 mg/kg) at 2 h post-APAP. Liver tissue was collected 2 h after the second NAC-S2 injection for quantification of protein-bound Cys-*S*-APAP adducts. Control groups included untreated mice and mice treated with APAP alone (without NAC-S2). Protein-bound Cys-*S*-APAP adducts were not detected in the untreated group. (C) and (D) show the effects of a single NAC-S2 administration at 30 min after APAP injection at 10 mg/kg and 20 mg/kg, respectively. Statistical analysis was performed using one-way ANOVA followed by Tukey's post hoc multiple comparisons test. Data were expressed as means ± SD (n ≥ 3). ∗*P* < 0.05, ∗∗*P* < 0.01, ∗∗∗*P* < 0.001.

NAPQI that is not detoxified binds covalently to protein thiols, leading to protein dysfunction and hepatocellular toxicity [[32], [33], [34]]. To assess this, we extracted proteins from APAP-treated mice liver according to the method shown in Fig. 7A, enzymatically digested the proteins, and quantified protein-bound Cys-*S*-APAP adducts. APAP administration markedly increased protein-bound Cys-*S*-APAP levels (Fig. 7B–D; Supplementary Fig. S10). However, in mice treated with NAC-S2 following APAP overdose, Cys-*S*-APAP levels were significantly reduced. These results demonstrate that NAC-S2 administration effectively suppresses the formation of APAP adducts on proteins associated with APAP overdose–induced liver injury. Cysteine persulfide is also present on protein thiol groups, and its levels may increase following NAC-S2 administration. In addition, NAPQI is presumed to form adducts with protein-bound CysSSH. Therefore, we also analyzed protein-bound Cys-*S*_*2*_-APAP. However, protein-bound Cys-*S*_*2*_-APAP was not detected in the samples prepared in this study (Supplementary Fig. S10). It is possible that the adduct was lost during the sample preparation process. Further optimization of the experimental conditions will be required for future analyses.

### 3.5 Protection of APAP-induced liver injury by supersulfide donors

**Fig. 8:**
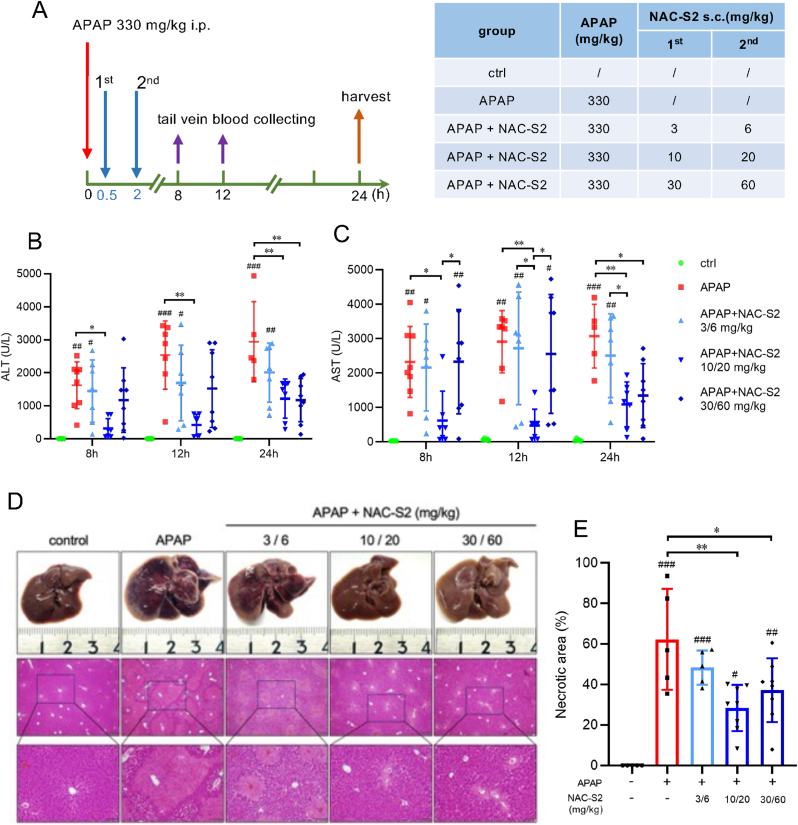
**Effects of NAC-S2 treatment on APAP-induced liver injury in mice.** (A) Experimental protocol. Mice were intraperitoneally administered APAP (330 mg/kg), followed by subcutaneous administration of NAC-S2 at 30 min and 2 h post-APAP at three different doses as indicated. Blood was collected at 8, 12, and 24 h post-APAP, and livers were harvested at 24 h post-APAP. (B, C) Time-course of serum ALT and AST levels. (D) Macroscopic appearance and H&E staining of liver tissues. (E) Quantification of necrotic area. Statistical analysis was performed using one-way ANOVA followed by Tukey's post hoc multiple comparisons test. Data were expressed as means ± SD (n ≥ 5). ∗*P* < 0.05, ∗∗*P* < 0.01, ∗∗∗*P* < 0.001; ^#^*P* < 0.05, ^# #^*P* < 0.01, ^# # #^*P* < 0.001 compared to control group.

We next examined whether the supersulfide-mediated enhancement of APAP detoxification confers protection against APAP-induced liver injury. Mice received subcutaneous NAC-S2 administration at the time points indicated in Fig. 8A following APAP injection. Serum samples were collected at 8, 12, and 24 h post-APAP administration to measure ALT and AST as markers of liver injury [35]. Liver tissue was also collected at 24 h for histopathological analysis. As shown in Fig. 8B and C, substantial increases in serum ALT and AST were observed 8 h after APAP injection, indicating significant hepatocellular damage occurred in this early stage. Notably, administration of NAC-S2 at 10 mg/kg (30 min post-APAP) and 20 mg/kg (2 h post-APAP) led to the most pronounced reduction in serum transaminase levels. These results demonstrate that NAC-S2 exerts protective effects against APAP-induced liver injury, particularly during the early stages.

The effects of NAC-S2 alone on serum transaminase levels are shown in Fig. S11. Although a slight increase in serum transaminase levels were observed following NAC-S2 administration at doses of 10/20 or 30/60 mg/kg, the extent was negligible. These results indicate that, under the present experimental conditions, NAC-S2 itself does not induce hepatotoxicity. While higher doses of NAC-S2 did not cause liver injury, they also did not significantly enhance its hepatoprotective effect against APAP-induced liver damage. Further investigation to determine the optimal therapeutic dose will be an important objective of future studies.

Histopathological examination also supported the protection effect of NAC-S2. H&E stained liver sections are shown in Fig. 8D. Compared to non-treated controls, APAP-overdosed mice exhibited pronounced histopathological changes, including centrilobular necrosis. These pathological alterations were markedly attenuated in mice administered NAC-S2 at doses of 10 mg/kg and 20 mg/kg. Quantification of necrotic areas (Fig. 8E) confirmed the histological evidence of NAC-S2-mediated hepatoprotection.

We also analyzed the effects of NAC-S2 on the extent of inflammation and macrophage dynamics in mouse livers after APAP administration. Specifically, MPO was examined as a marker of inflammation, and macrophage numbers were assessed immunohistochemically using F4/80 as a pan-macrophage marker. As shown in Supplementary Fig. S12, APAP administration resulted in a significant increase in the number of MPO-positive cells in the liver, confirming the induction of inflammation. Treatment with NAC-S2 did not significantly affect the number of MPO-positive cells. The number of F4/80-positive cells was not changed after APAP administration compared with the control. NAC-S2 treatment increased the number of macrophages following APAP administration. One possible explanation for these observations is that hepatic damage had already progressed 24 h after APAP administration, with extensive expansion of necrotic areas in the liver (Fig. 8D and E). As described above, NAC-S2 treatment reduced the necrotic area (Fig. 8D and E). Because the numbers of MPO-positive and F4/80-positive cells were normalized to the total liver area, the increase in viable (non-necrotic) liver tissue following NAC-S2 treatment may have led to an apparent increase in cell counts, thereby masking the anti-inflammatory effect of NAC-S2. To more accurately evaluate the effect of NAC-S2 on APAP-induced inflammation, analyses at an earlier time point, before the appearance of hepatic necrotic areas, may be required.

### 3.6 Anti-inflammatory actions of supersulfide donors against APAP-induced liver injury

**Fig. 9:**
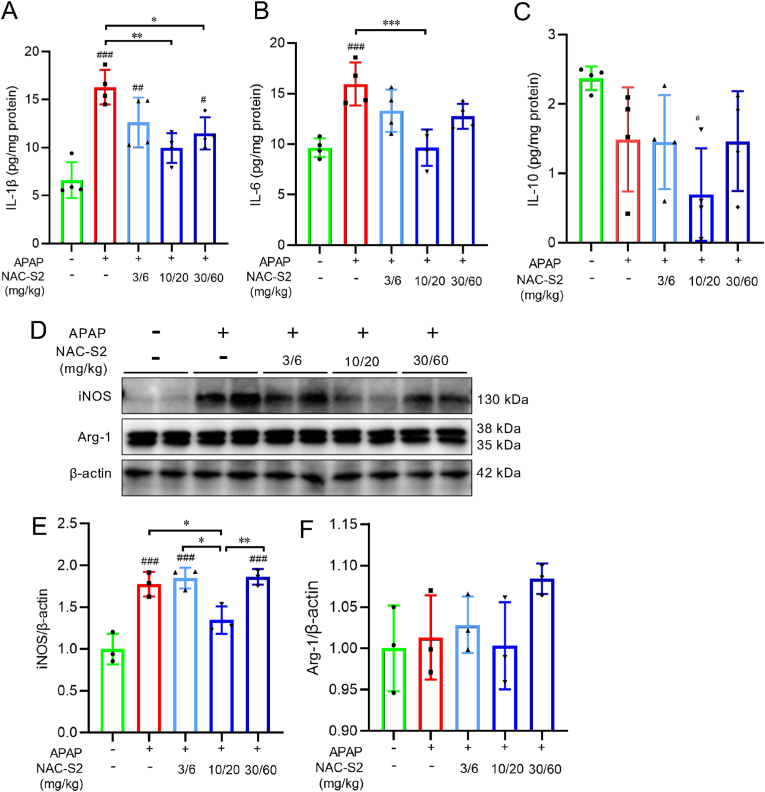
**Suppression of proinflammatory cytokine production and M1-type macrophage polarization by a supersulfide donor in livers from APAP-treated mice.** (A–C) Levels of IL-1β, IL-6, and IL-10 in liver tissue. Mice were intraperitoneally administered APAP (330 mg/kg), followed by subcutaneous NAC-S2 administration at 30 min and 2 h post-APAP. Livers were harvested 24 h after APAP administration, and cytokine levels were quantified by ELISA. (D) Western blot analysis of hepatic iNOS and Arg-1 expression. (E, F) Quantification of Western blot band intensities. Relative protein levels were normalized to β-actin. Uncropped blots are shown in Supplementary Fig. S17. Statistical analysis was performed using one-way ANOVA followed by Tukey's post hoc multiple comparisons test. Data were expressed as means ± SD (n ≥ 3). ∗*P* < 0.05, ∗∗*P* < 0.01, ∗∗∗*P* < 0.001; ^#^*P* < 0.05, ^# #^*P* < 0.01, ^# # #^*P* < 0.001 compared to control group.

Acute liver injury induced by APAP serves as a trigger for recruitment and activation of inflammatory cells, which exacerbate hepatic damage [6,7]. As shown in Fig. 9, 24 h post-APAP administration, levels of pro-inflammatory cytokines IL-1β and IL-6 were significantly elevated in liver tissues. NAC-S2 treatment suppressed this increase, suggesting both a direct detoxification effect through supersulfides and an anti-inflammatory action intrinsic to NAC-S2. This was further supported by the observation that IL-1β and IL-6 levels remained suppressed even at higher doses of NAC-S2 (30 mg/kg and 60 mg/kg).

To further characterize the inflammatory responses, hepatic macrophage polarization was assessed by evaluating M1 and M2 markers, iNOS and Arg-1, respectively. APAP administration resulted in a pronounced increase in iNOS expression, indicating M1 macrophage polarization. NAC-S2 significantly suppressed iNOS induction, while Arg-1 expression remained unaffected by either APAP or NAC-S2 treatment. These findings indicate that NAC-S2 suppresses the inflammatory responses associated with APAP-induced liver injury and exerts protective effects even in the later stages of hepatotoxicity.

When protein adduct formation by NAPQI occurs on mitochondrial proteins, mitochondrial function—particularly the electron transport chain—is impaired, leading to enhanced production of reactive oxygen species (ROS) from mitochondria [36]. As shown in Fig. 9, hepatic expression of iNOS is markedly increased in mice administered an overdose of APAP. As a result, excessive amounts of ROS and NO are produced, leading to the generation of reactive nitrogen species (RNS), including peroxynitrite, which are thought to further exacerbate mitochondrial dysfunction [36]. Therefore, we analyzed 3-nitrotyrosine as a marker of RNS generation. As shown in Supplementary Fig. S13, APAP overdose significantly increased 3-nitrotyrosine levels in liver proteins. Furthermore, administration of NAC-S2 reduced the APAP-induced increase in 3-nitrotyrosine to levels comparable to those of the control group. These results indicate that NAC-S2 treatment suppresses oxidative stress induced by APAP overdose.

Oxidative stress induced by APAP overdose is known to activate the transcription factor Nrf2, thereby upregulating the expression of various antioxidant, anti-inflammatory, and detoxifying enzyme genes [37]. To investigate the effect of NAC-S2 on Nrf2 activation, we examined the expression of glutamate–cysteine ligase catalytic subunit (GCLC), an Nrf2-regulated gene. Under the experimental conditions used in this study, APAP overdose did not induce a significant increase in GCLC expression. In contrast, although NAC-S2 treatment suppressed oxidative stress in the liver, it significantly increased GCLC expression (Supplementary Fig. S14). These results suggest that NAC-S2 treatment may activate Nrf2-mediated gene expression, thereby contributing, at least in part, to its hepatoprotective effects against APAP-induced liver injury.

Previous studies have reported that intraperitoneal administration of high-dose NAC (e.g., 200 mg/kg) attenuates liver injury caused by APAP overdose [38]. Therefore, in this study, we analyzed the effects of high-dose NAC treatment on APAP-induced liver damage. As shown in Supplementary Fig. S15, subcutaneous administration of NAC at a high dose reduced the levels of the liver injury markers ALT and AST. In addition, the production of inflammatory cytokines in the liver induced by APAP overdose was also suppressed. Taken together, these results confirm that NAC itself exerts a protective effect against APAP-induced liver injury, consistent with previous reports, and further demonstrate that NAC-S2 exhibits superior therapeutic efficacy at doses less than one-tenth of that of NAC.

**Fig. 10:**
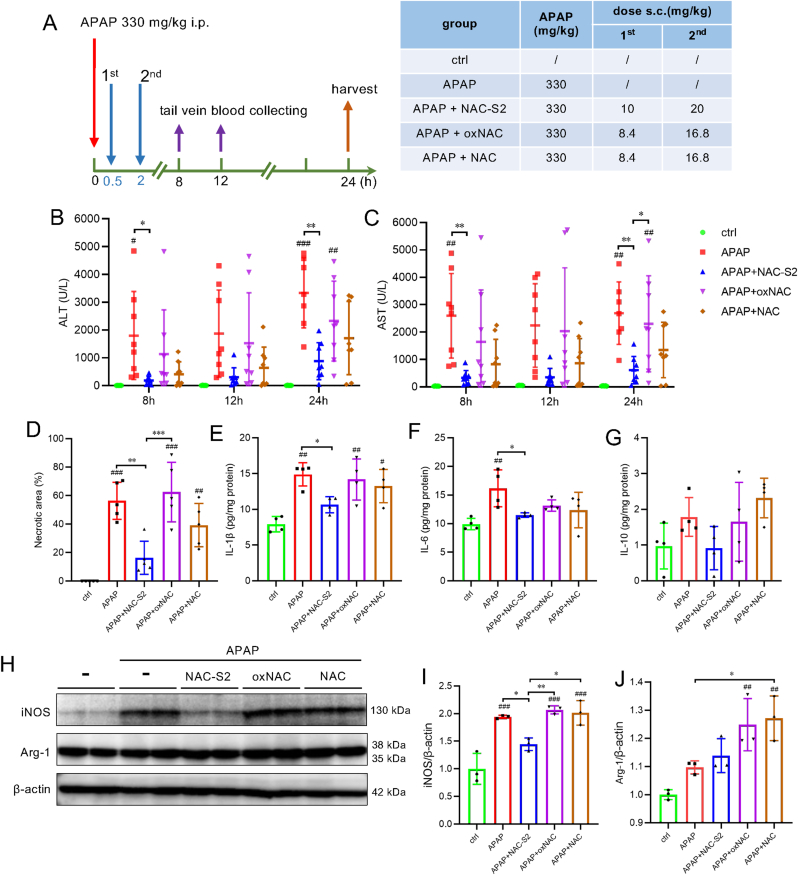
**Effects of NAC-S2, oxNAC, and NAC on APAP-induced liver injury.** (A) Experimental protocol. Mice were intraperitoneally administered APAP (330 mg/kg), followed by subcutaneous administration of sulfur compounds (NAC-S2, oxNAC, or NAC) at 30 min and 2 h post-APAP. Blood was collected at 8, 12, and 24 h post-APAP, and liver tissue was harvested at 24 h post-APAP. (B, C) Time-course of serum ALT and AST levels. (D) Quantification of necrotic area in liver. Macroscopic appearance and H&E staining of liver tissues are shown in Supplementary Fig. S16 (E–G) Hepatic cytokine levels: IL-1β (E), IL-6 (F), and IL-10 (G). (H) Western blot analysis of iNOS and Arg-1 expression in liver. (I, J) Quantification of Western blot band intensities. Relative protein levels were normalized to β-actin. Uncropped blots are shown in Supplementary Fig. S18. Statistical analysis was performed using one-way ANOVA followed by Tukey's post hoc multiple comparisons test. Data were expressed as means ± SD (n ≥ 3). ∗*P* < 0.05, ∗∗*P* < 0.01, ∗∗∗*P* < 0.001; ^#^*P* < 0.05, ^# #^*P* < 0.01, ^# # #^*P* < 0.001 compared to control group.

To confirm that the protective effect of NAC-S2 is due to its supersulfide-donating capability, we compared it to oxNAC, a control compound lacking such activity [25,28,29,39]. Additionally, we evaluated whether the observed effect could be attributed to N-acetylcysteine, which has previously been reported to have hepatoprotective effects against APAP toxicity [1]. As shown in Fig. 10 and S16, only NAC-S2, and not oxNAC or NAC (at equivalent doses), conferred protection against APAP-induced liver injury, indicating that the protective effect is specific to supersulfide donation.

**Fig. 11:**
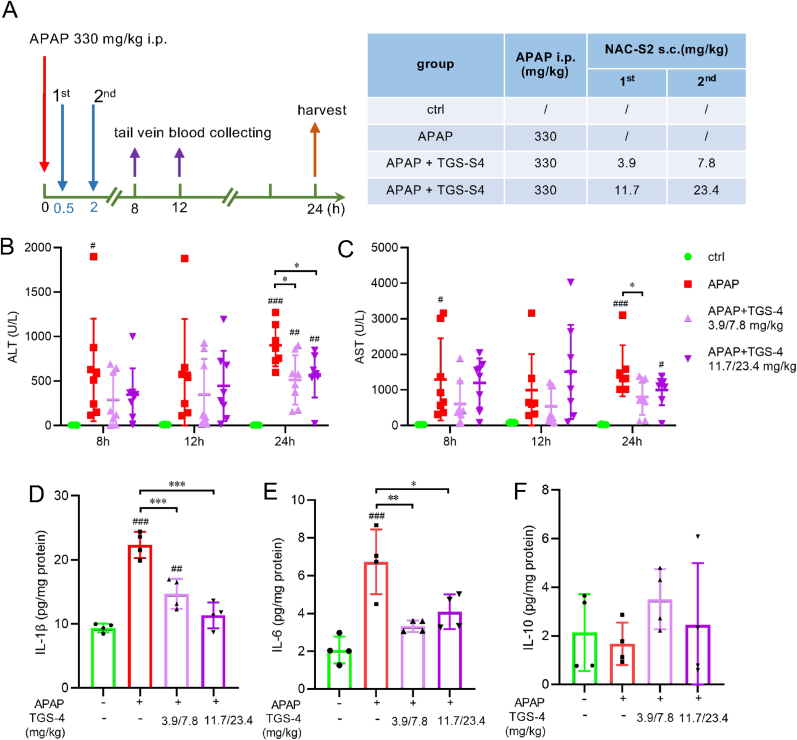
**Effects of the supersulfide donor TGS4 on APAP-induced liver injury.** (A) Experimental protocol. Mice were intraperitoneally administered APAP (330 mg/kg), followed by subcutaneous injection of TGS4 at 30 min and 2 h post-APAP. Two different doses were used as indicated. Blood was collected at 8, 12, and 24 h post-APAP, and liver tissue was harvested at 24 h post-APAP. (B, C) Time-course of serum ALT and AST levels. (D–F) Hepatic cytokine levels: IL-1β (D), IL-6 (E), and IL-10 (F). Statistical analysis was performed using one-way ANOVA followed by Tukey's post hoc multiple comparisons test. Data were expressed as means ± SD (n ≥ 4). ∗*P* < 0.05, ∗∗*P* < 0.01, ∗∗∗*P* < 0.001; ^#^*P* < 0.05, ^# #^*P* < 0.01, ^# # #^*P* < 0.001 compared to control group.

Finally, we investigated the effect of another supersulfide donor, TGS4 [26,39]. As illustrated in Fig. 11, TGS4 administration significantly reduced serum ALT and AST levels and suppressed IL-1β and IL-6 production in the APAP overdose model. Taken together, these findings suggest that increasing hepatic supersulfide levels not only attenuates the acute phage of liver injury but also suppresses the subsequent inflammatory responses, highlighting the therapeutic potential of supersulfide donors in the treatment of APAP-induced hepatotoxicity.

## 4 Discussion

The primary treatment for APAP poisoning is the administration of the specific detoxifying agent NAC, which mitigates APAP-induced hepatic injury [1]. In cases of acute liver failure, liver transplantation is also considered as a last-resort intervention [1,40]. However, the clinical use of NAC treatment is sometimes limited by adverse effects and the potential for treatment resistance [1]. Therefore, the development of novel therapeutic agents with improved efficacy is urgently needed [1,2,41].

**Fig. 12:**
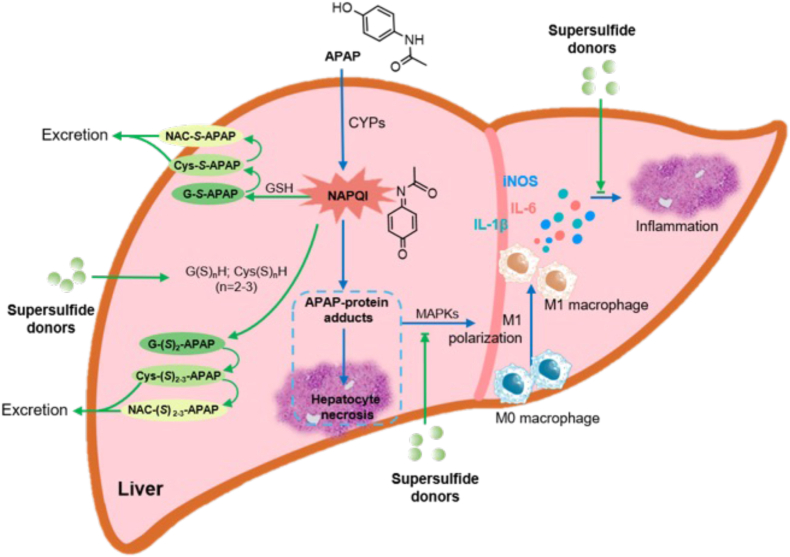
**Schematic diagram of supersulfide donors alleviating APAP-induced liver injury.** APAP is metabolized in the liver by cytochrome P450 enzymes (CYPs) to form *N*-acetyl-*p*-benzoquinone imine (NAPQI), a highly electrophilic metabolite. NAPQI readily conjugates with intracellular glutathione (GSH), forming glutathione adducts that are subsequently metabolized to cysteine conjugates and excreted in the urine. During this process, hepatic GSH levels become depleted. As a result, excess NAPQI that cannot be detoxified by GSH binds covalently to cellular proteins, leading to protein dysfunction and cytotoxicity. Supersulfide donors rapidly transfer sulfur atoms to hepatic GSH and cysteine, thereby increasing the levels of corresponding supersulfide species. These supersulfides exhibit enhanced nucleophilicity, enabling them to detoxify NAPQI more efficiently than GSH alone. In addition to promoting detoxification, supersulfide donors markedly suppress inflammation associated with liver injury. Notably, they inhibit the differentiation of macrophages into the pro-inflammatory M1 phenotype.

In the present study, we demonstrated that supersulfide donors exerted hepatoprotective effects against APAP-induced liver injury in a murine model. As illustrated in Fig. 12, supersulfide donors appear to confer multifaceted protection against APAP-induced hepatotoxicity. The supersulfide donor NAC-S2 showed superior hepatoprotective effects compared to NAC in the context of APAP overdose (Fig. 10). Notably, NAC did not exhibit significant protective effects in our study, likely due to the substantially lower dosage (10 mg/kg and 20 mg/kg, subcutaneous injection) and different route of administration compared to previously reported studies (e.g., 200 mg/kg, intraperitoneal injection) [38]. Nevertheless, even at these lower doses, NAC-S2 provided sparked therapeutic benefits. Consistent with previous reports [38], increasing the dose of NAC to more than ten times that of NAC-S2 resulted in a protective effect against APAP overdose–induced liver injury (Supplementary Fig. S15).

One plausible mechanism underlying the protective effects of supersulfide donors involves their ability to increase hepatic supersulfide levels, which in turn facilitates detoxification of the reactive APAP metabolite NAPQI. Urinary analysis of mice subjected to APAP overdose confirmed the excretion of the NAPQI–GSH conjugate (G-*S*-APAP), its downstream metabolites Cys-*S*-APAP and NAC-*S*-APAP, as well as their corresponding hydropersulfide derivatives. In mice treated with NAC-S2, these conjugates were markedly increased, suggesting that the reactions of NAPQI with GSH and GSSH in the liver were enhanced. Notably, NAC-*S*-APAP and NAC-*S*_*2*_-APAP were substantially elevated following NAC-S2 administration. As shown in Fig. 3, subcutaneously administered NAC-S2 transferred sulfur atoms to GSH and cysteine, while being converted to oxNAC, which subsequently circulated in the bloodstream. Under these conditions, small amounts of NAC or NAC hydropersulfide were detected in the circulation. Furthermore, in vitro experiments using HepG2 cells demonstrated that treatment with oxNAC did not significantly increase NAC-*S*_*2*_-APAP or NAC-*S*_*3*_-APAP levels. Although NAC-*S*_*2*_-APAP and NAC-*S*_*3*_-APAP increased in HepG2 cells treated with NAC-S2, this effect is likely attributable to the relatively high concentrations of NAC-S2 present under in vitro conditions. Taken together, the marked increase in NAC-*S*-APAP and NAC-*S*_*2*_-APAP observed in APAP-overdosed mice following NAC-S2 administration is unlikely to result directly from circulating NAC-S2. Rather, it is more plausibly explained by increased formation of their precursor conjugates—G-*S*-APAP and Cys-*S*-APAP—as well as their hydropersulfide adducts, whose production appears to be enhanced by NAC-S2 treatment.

It has been reported that when intracellular GSH is sufficiently available at millimolar concentrations, the direct non-enzymatic reaction between NAPQI and GSH predominates [42]. However, during APAP overdose, intracellular GSH levels are markedly depleted and can decrease to submillimolar levels. Under such conditions, glutathione S-transferase (GST) is thought to enzymatically facilitate the reaction between NAPQI and GSH by lowering the pKa of GSH [42]. In contrast, intracellular GSSH is present at only a few percent of total GSH (Fig. 4), with tissue concentrations of approximately 0.1 mM in the liver. Notably, the reported pKa of GSSH (5.4–6.9) [43,44] is comparable to that of GSH when bound to GST (5.2–6.8) [45]. Therefore, even at these lower concentrations, GSSH is likely capable of directly reacting with NAPQI. To our knowledge, there have been no reports examining whether GST binds to GSSH or how such binding might affect its pKa.

Interestingly, mice lacking GST Pi (GSTP), the GST isoform that most strongly catalyzes the glutathione conjugation of APAP [42], have been reported to be resistant to liver injury induced by APAP overdose [46]. Subsequent studies demonstrated that hepatic protein S-glutathionylation after APAP administration is enhanced in GSTP-deficient mice compared with wild-type mice [47]. Furthermore, it has been suggested that this increase in protein S-glutathionylation may induce an adaptive response to APAP overdose, thereby exerting a protective effect against liver injury [47]. Elucidating how NAC-S2 administration affects hepatic protein persulfidation in mice subjected to APAP overdose is therefore important, particularly in relation to the hepatoprotective effects of NAC-S2. In the present study, we analyzed protein-bound Cys-*S*_*2*_-APAP as a marker of protein persulfidation (Supplementary Fig. S10); however, these adducts were not detected in the samples analyzed. Future studies will be required to further investigate the post-translational modification status of protein thiol groups following NAC-S2 administration, including direct assessment of protein persulfidation.

NAC-S2 treatment prevented the depletion of hepatic GSH, typically observed following APAP overdose (Fig. 5), and reduced the formation of protein-bound APAP, a key event in APAP hepatotoxicity (Fig. 7). Previous studies have shown that NAPQI preferentially binds to mitochondrial proteins [34], and excessive mitochondrial NAPQI adduct formation can disrupt the electron transport chain, promoting reactive oxygen species generation, DNA fragmentation, and ultimately cell death [34,48]. Supersulfide donor treatment preserved hepatic GSH levels and suppressed NAPQI-protein adduct formation, thereby likely contributing to the inhibition of mitochondrial damage and subsequent cell death. Indeed, treatment with NAC-S2 significantly reduced the necrotic area in the livers of APAP-treated mice (Fig. 8 and S16).

In the analysis of urinary APAP and its metabolites, variability in quantitative values was observed between experiments. Although the same dose of APAP was administered in Fig. 6B and C, differences were detected in the amounts of urinary metabolites. One possible explanation for this discrepancy is the timing of the experiments, such as whether urine samples were collected in the morning or in the evening. It is well known that the concentration of urinary metabolites varies depending on the rate of water reabsorption in the kidneys [49]. To account for this variability, urinary creatinine is commonly used for normalization. Therefore, future studies should examine normalization of APAP-supersulfide adduct levels using urinary creatinine. On the other hand, in the present study, urine collection for comparisons under identical experimental conditions was performed at the same time points. Thus, when comparing groups with and without NAC-S2 administration, the influence of urine volume is likely minimal, and reproducibility was confirmed.

Recent studies also suggested that GSH depletion in the liver during APAP overdose may trigger ferroptosis, and iron-dependent form of regulated cell death characterized by lipid peroxide accumulation [50,51]. Intriguingly, hydropersulfides, a class of supersulfides, have been shown to effectively scavenge lipid peroxyl radicals, thereby strongly suppressing lipid peroxidation [22,[52], [53], [54]]. Thus, supersulfide donors may not only replenish GSH levels but also attenuate the accumulation of lipid peroxides, offering a dual mechanism of protection. Further investigation is warranted to elucidate this possibility.

In addition to direct cellular damage by NAPQI, excessive inflammatory responses are known to exacerbate APAP-induced liver injury [1,6,7]. Anti-inflammatory effects have also been implicated in the therapeutic action of NAC [55]. In our study, administration of supersulfide donors attenuated the hepatic inflammatory response. We previously reported that NAC-S2 blocks the NF-κB pathway and robustly inhibits pro-inflammatory cytokine production, with efficacy surpassing that of NAC [[23], [24], [25], [26], [27]]. These findings suggest that the anti-inflammatory properties of supersulfide donors contribute to their hepatoprotective effects against APAP overdose.

Using mass spectrometry, we successfully identified conjugates between NAPQI and supersulifdes, providing direct evidence for their interaction in vivo. In addition, we established a sensitive MRM method to quantify supersulfide-NAQPI conjugates excreted in urine. In this study, standard curves were generated using authentic standards prepared before and after sample analysis. For more precise quantification, it will be necessary to develop an isotope-dilution method using stable isotope-labeled standards spiked into the samples [19].

While supersulfide donors represent a promising approach for increasing hepatic supersulfide levels, further optimization of dosing and administration route is necessary before clinical application. Moreover, adverse effects associated with NAC therapy–including allergic reactions, nausea, vomiting, gastrointestinal disturbances, fever, headache, drowsiness, and hypotension–have been reported, particularly with intravenous administration [56]. Thorough evaluation of the safety and potential side effects of supersulfide donors is essential for their clinical development.

Ongoing efforts are exploring alternative therapeutic strategies for APAP overdose [1,2]. As APAP is bioactivated to toxic NAPQI via CYP2E1, inhibitors of this enzyme may attenuate toxicity. For example, fomepizole, a CYP2E1 inhibitor, has been shown to alleviate APAP-induced hepatotoxicity when co-administered with NAC [57]. These findings raise the possibility that combining CYP2E1 inhibitors with supersulfide donors may offer enhanced protection against APAP overdose.

The transcription factor nuclear fact erythroid 2-related factor 2 (Nrf2) is known to induced antioxidant and detoxifying enzymes, providing protection against APAP-induced liver injury [58,59]. Upon activation, Nrf2 upregulates the expression of the cystine transporter xCT, enhancing cystine uptake and increasing intracellular cysteine availability [60]. Takeda et al. reported that Nrf2-xCT pathway activation raises intracellular supersulfide levels via enhancing cysteine metabolism [61]. These finding suggest that the hepatoprotective effects of previously reported Nrf2 activators may be partly mediated by increased supersulfide production through xCT induction. Interestingly, NAC-S2 enhanced the expression of Nrf2-dependent genes in the livers of mice subjected to APAP overdose (Supplementary Fig. S14). Nrf2 is known to exert protective effects against APAP overdose–induced liver injury by inducing the expression of various antioxidant and detoxifying enzymes [37]. Therefore, Nrf2 activators are expected to have therapeutic potential for APAP-induced liver injury. In the present study, NAC-S2 administration was suggested to increase hepatic Nrf2 activity; however, the underlying mechanism remains to be elucidated. Some groups have reported that persulfidation of cysteine residues in Keap1, a negative regulator of Nrf2, promotes its dissociation from Nrf2, thereby resulting in Nrf2 activation [62,63]. Further studies are required to determine whether NAC-S2 activates Nrf2 through the induction of Keap1 persulfidation.

In conclusion, our study is the first to demonstrate that hepatic supersulfides directly conjugate with NAPQI to form supersulfide-APAP adducts. We showed that administration of supersulfide donors markedly enhances detoxification of NAPQI, reduces hepatic inflammation, and protects against APAP-induced liver injury. These findings support the development of supersulfide-targeted therapies as a novel strategy for the treatment of APAP overdose.

## CRediT authorship contribution statement

**Chunyu Guo:** Data curation, Investigation, Writing – original draft. **Touya Toyomoto:** Investigation. **Hiroyasu Tsutsuki:** Investigation. **Yukio Fujiwara:** Investigation. **Yoshihiro Komohara:** Investigation. **Tianli Zhang:** Investigation. **Hina Honda:** Investigation. **Stephen Lindahl:** Investigation. **Takuro Niidome:** Investigation. **Jun Fang:** Investigation. **Ming Xian:** Investigation. **Tomohiro Sawa:** Conceptualization, Funding acquisition, Investigation, Supervision, Writing – original draft.

## Declaration of competing interest

The authors declare that they have no known competing financial interests or personal relationships that could have appeared to influence the work reported in this paper.

## Abbreviations

ALT alanine aminotransferase.

APAP acetaminophen.

Arg-1 arginase-1.

AST aspartate aminotransferase.

CYP cytochrome P450.

CysSH cysteine.

CysSSH cysteine hydropersulfide.

Cys-*S*-APAP cysteine-APAP adduct.

Cys-*S*_*2*_-APAP CysSSH-APAP adduct.

Cys-*S*_*3*_-APAP cysteine hydrotrisulfide APAP adduct.

GCLC glutamate–cysteine ligase catalytic subunit.

GSH glutathione.

GSSH glutathione hydropersulfide.

GSSSH glutathione hydrotrisulfide.

G-*S*-APAP GSH-APAP adduct.

G-*S*_*2*_-APAP GSSH-APAP conjugates.

GST Glutathione S-transferases.

HPLC high performance liquid chromatography.

H&E hematoxylin and eosin

iNOS inducible nitric oxide synthase.

LC-MS/MS liquid chromatography-tandem mass spectrometry.

MRM multiple reaction monitoring.

NAC N-acetylcysteine.

NAC-S2 N-acetylcysteine tetrasulfide.

NAC-*S*-APAP NAC-APAP adduct.

NAC-*S*_*2*_-APAP NAC hydropersulfide APAP adduct.

NAC-*S*_*3*_-APAP NAC hydrotrisulfide APAP adduct.

NAPQI N-acetyl-p-benzoquinone imine.

Nrf2 nuclear fact erythroid 2-related factor 2

oxNAC oxidized NAC.

TGS4 thioglucose tetrasulfide.
