# Supplementary material for: Hepatic supersulfides attenuate acetaminophen-induced liver injury via enhanced detoxification and anti-inflammatory mechanisms
Source: Redox Biol. 2026 Mar 28;92:104140. doi: 10.1016/j.redox.2026.104140 (PMC13068582; doi:10.1016/j.redox.2026.104140)
Supplement: Multimedia component 1 [file mmc1.pdf]

Supplementary Table 1 MRM parameters for APAP adducts.

Analyte	Precursor ion (m/z)	Product ion (m/z)	Fragmentor voltage (V)	Collision energy (eV)	Polarity
APAP	152.1	110	90	13	+
G-*S*-APAP	457.1	140	130	45	+
G-S_2-APAP	489.1	360.1	90	13	+
Cys-*S*-APAP	271.1	139.9	90	25	+
Cys-S_2-APAP	303.1	184.1	90	17	+
Cys-S_3-APAP	335	184	90	13	+
NAC-*S*-APAP	313.1	208	90	13	+
NAC-S_2-APAP	345.1	140	90	37	+
NAC-S_3-APAP	377	171.9	90	33	+
CysS-HPE-AM	299	121	90	29	+
Cys^{34}S-HPE-AM	301	121	90	29	+
CysS-S-HPE-AM	331	121	50	33	+
Cys^{34}S-S-HPE-AM	333	121	50	33	+
GS-HPE-AM	485	356.2	90	13	+
[$^{13}C_2$,^{15}N]GS-HPE-AM	488	359.2	90	13	+
GSS-HPE-AM	517	388.2	50	17	+
[$^{13}C_2$,^{15}N]GSS-HPE-AM	520	391.2	50	17	+
GSSS-HPE-AM	549	419.9	50	17	+
[$^{13}C_2$,^{15}N]GSSS-HPE-AM	552	422.9	50	17	+
3-nitrotyrosine	227.1	180.7	90	9	+
Tyrosine	182.1	135.8	90	9	+
NAC-S-HPE	341.1	120.9	90	37	+
NAC-SS-HPE	373.1	120.8	130	37	+
oxNAC	325.1	162	103	17	+
NAC-S2	389	343.1	103	9	+

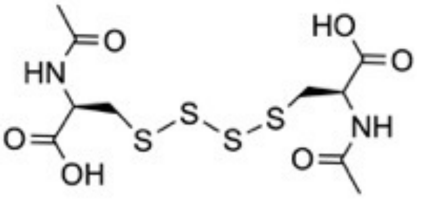

NAC-S2

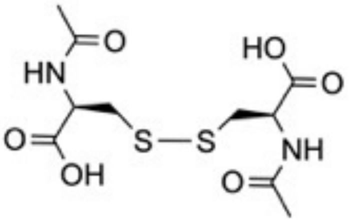

oxNAC

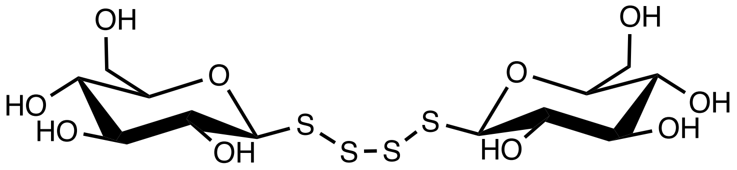

TGS4

Supplementary Fig. S1. Chemical structures of supersulfide donors used in this study. Chemical structure of oxNAC, that lacks sulfur donating capability, is also shown.

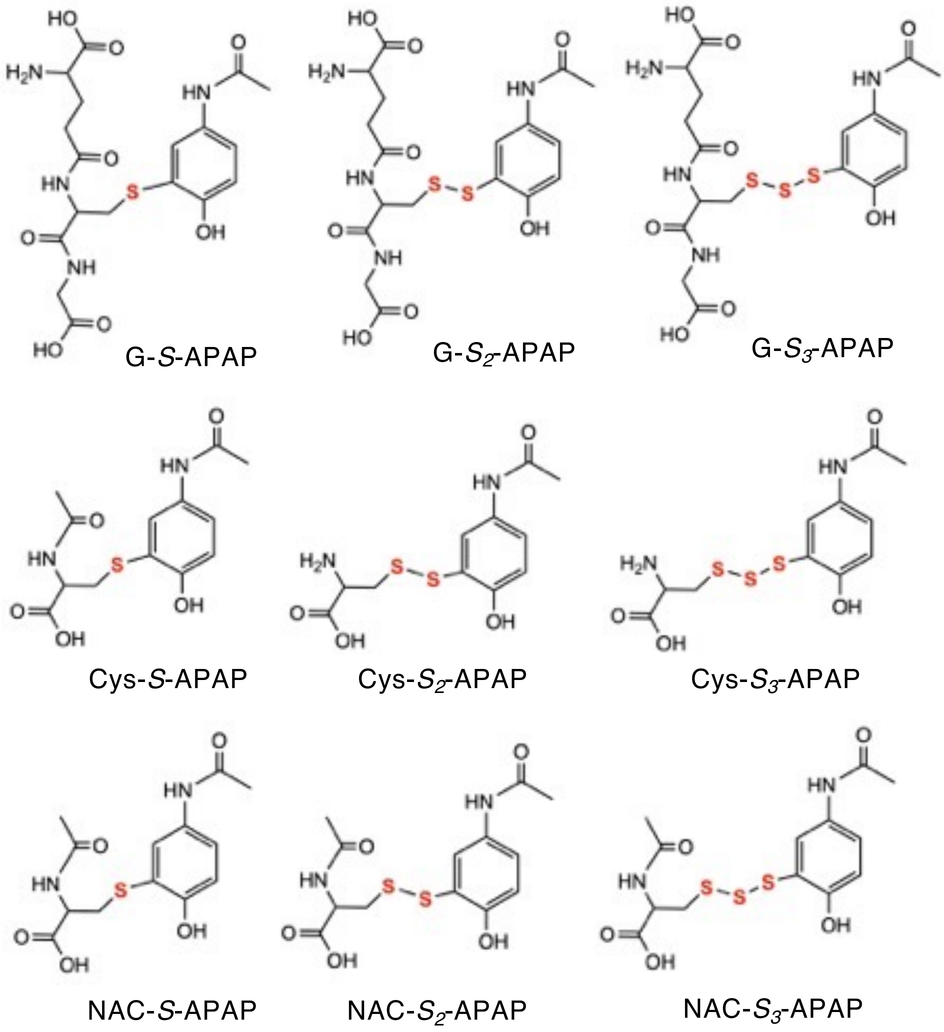

Supplementary Fig. S2. APAP conjugates analyzed in this study. Authentic standards of supersulfide-APAP conjugates were newly prepared in this study.

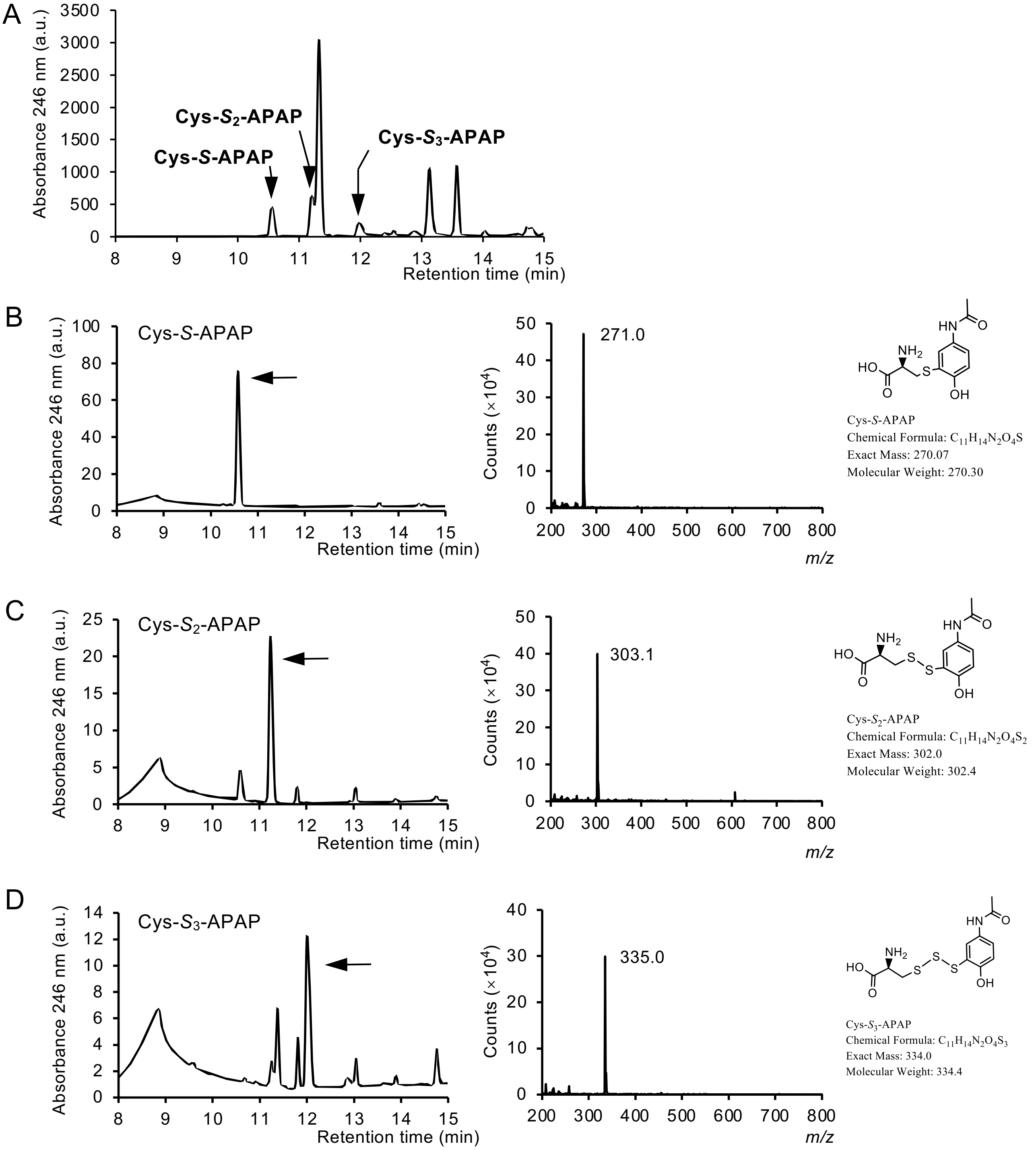

Supplementary Fig. S3. Characterization cysteine supersulfide-APAP adducts. (A) Reverse-phase HPLC separation for reaction mixture of cysteine, Na_2S_2, and NAPQI. Peaks correspond to Cys-S_n-APAP adducts (n, 1~3) indicated by arrows were collected and subjected for HPLC and mass spectrometry analyses. Characterization of (B) Cys-*S*-APAP, (C) Cys-S_2-APAP, and (D) Cys-S_3-APAP. Left panels show HPLC chromatograms, middle panels show mass chromatograms, and right panels show chemical structures.

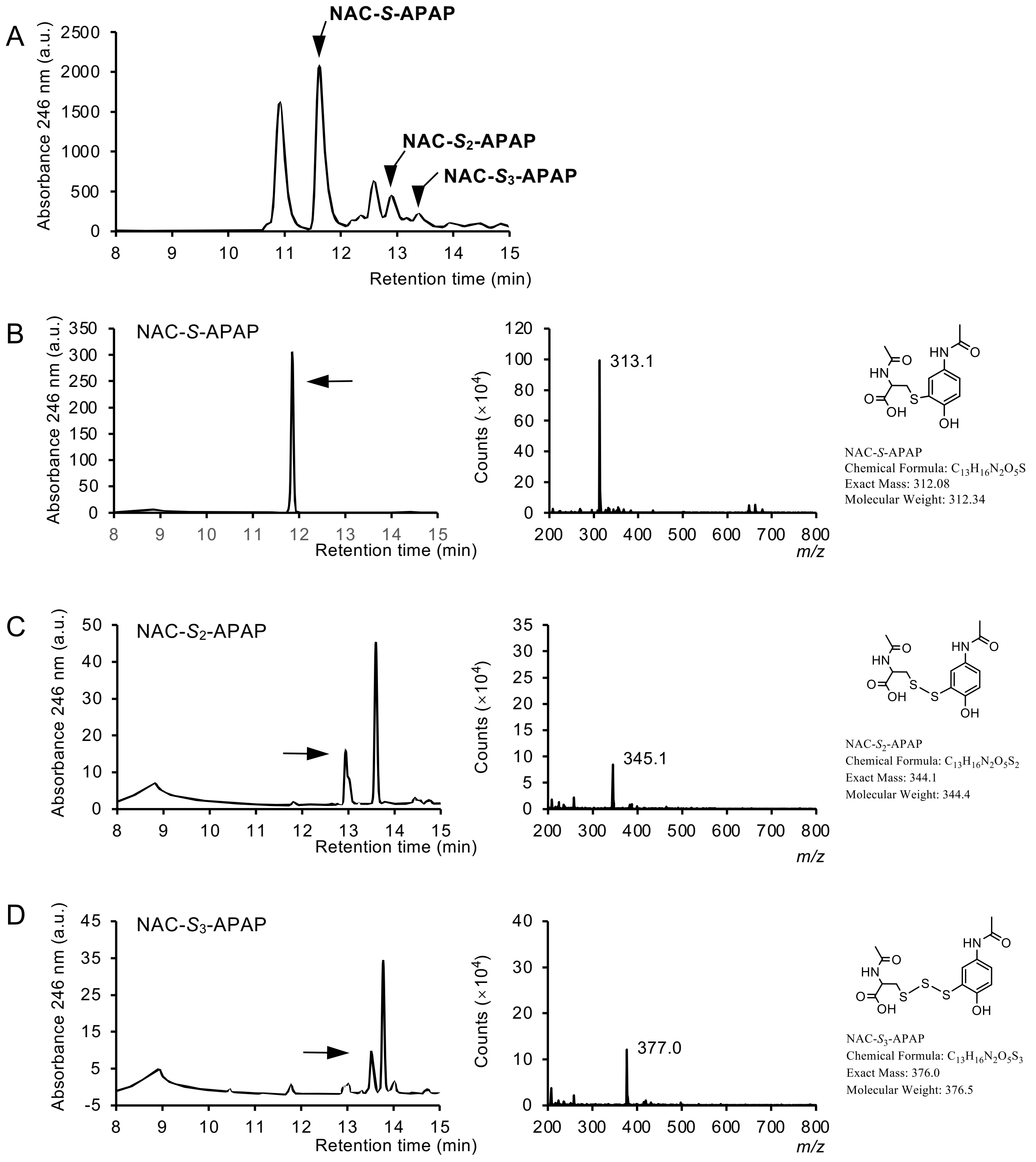

Supplementary Fig. S4. Characterization N-acetyl-L-cysteine supersulfide-APAP adducts. (A) Reverse-phase HPLC separation for reaction mixture of N-acetyl-L-cysteine, Na_2S_2, and NAPQI. Peaks correspond to NAC-S_n-APAP adducts (n, 1~3) indicated by arrows were collected and subjected for HPLC and mass spectrometry analyses. Characterization of (B) NAC-*S*-APAP, (C) NAC-S_2-APAP, and (D) NAC-S_3-APAP. Left panels show HPLC chromatograms, middle panels show mass chromatograms, and right panels show chemical structures.

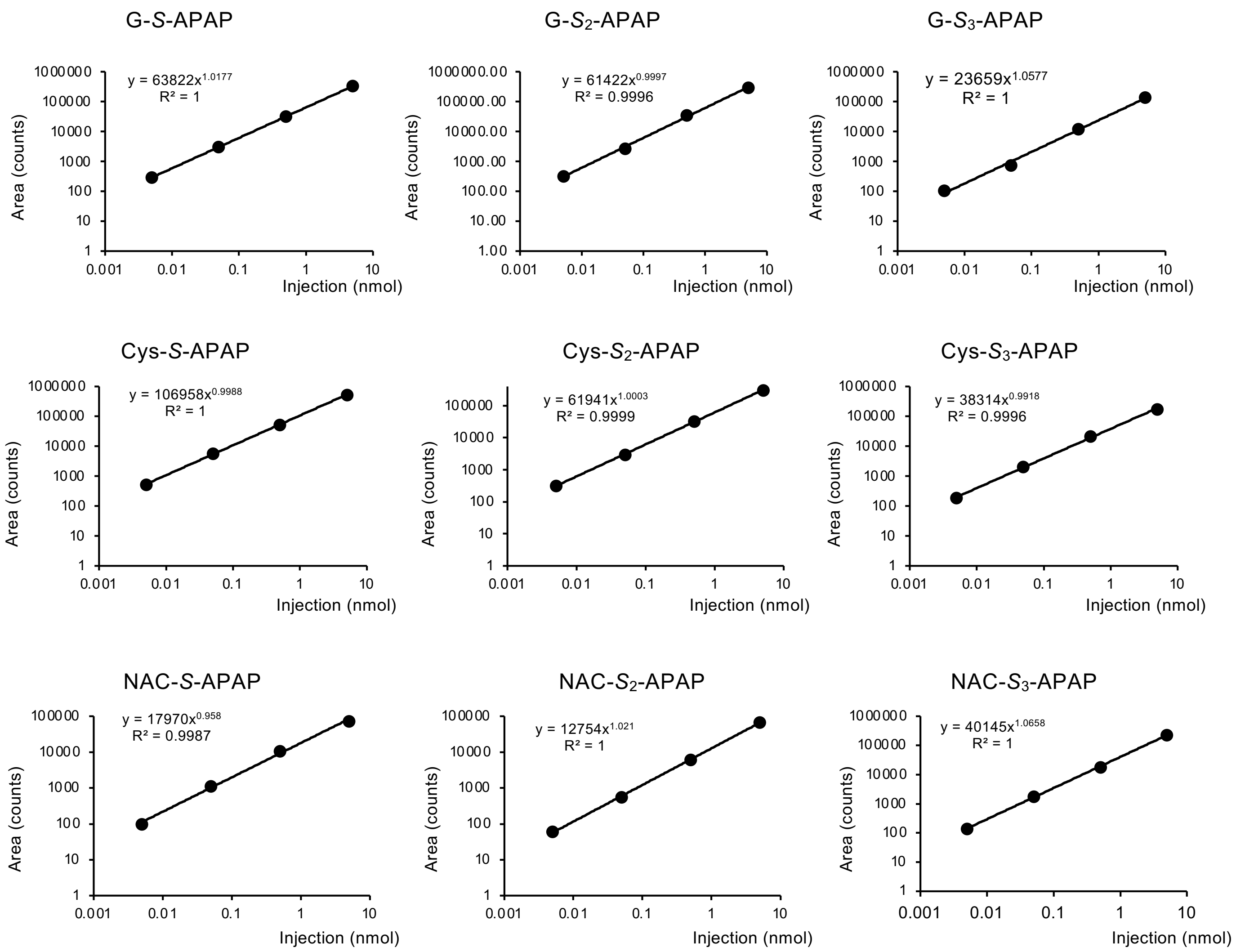

Supplementary Fig. S5. Calibration curves for APAP adducts determined by means of LC-MS/MS with MRM. MRM parameters used are shown in Supplementary Table S1.

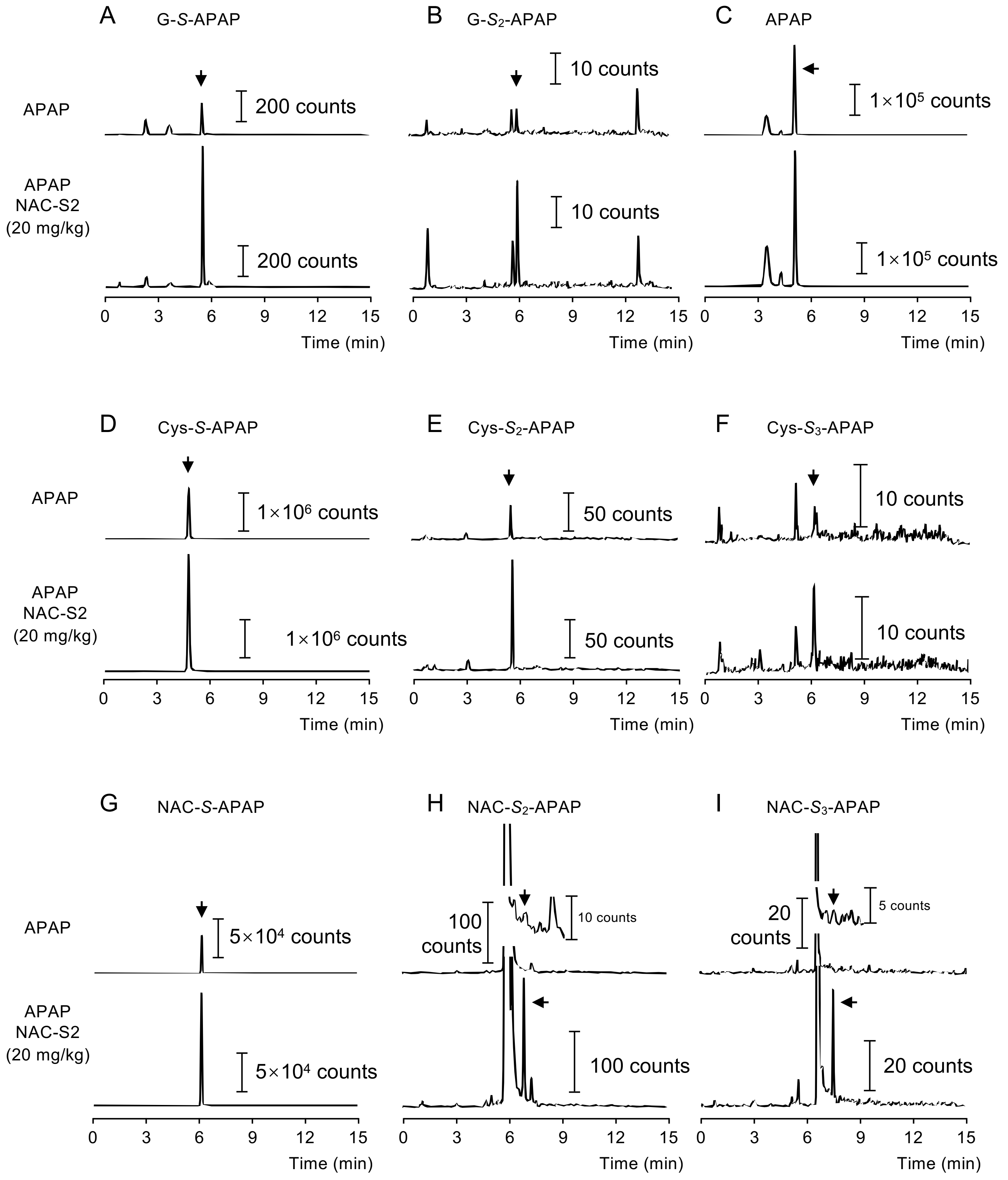

Supplementary Fig. S6. Representative MRM chromatograms of (A) G-*S*-APAP, (B) G-S_2-APAP, (C) APAP, (D) Cys-*S*-APAP, (E) Cys-S_2-APAP, (F) Cys-S_3-APAP, (G) NAC-*S*-APAP, (H) NAC-S_2-APAP, and (I) NAC-S_3-APAP from bladder urine sample of APAP injected mice and bladder urine sample of 20 mg/kg NAC-S2 treated on APAP-injected mice.

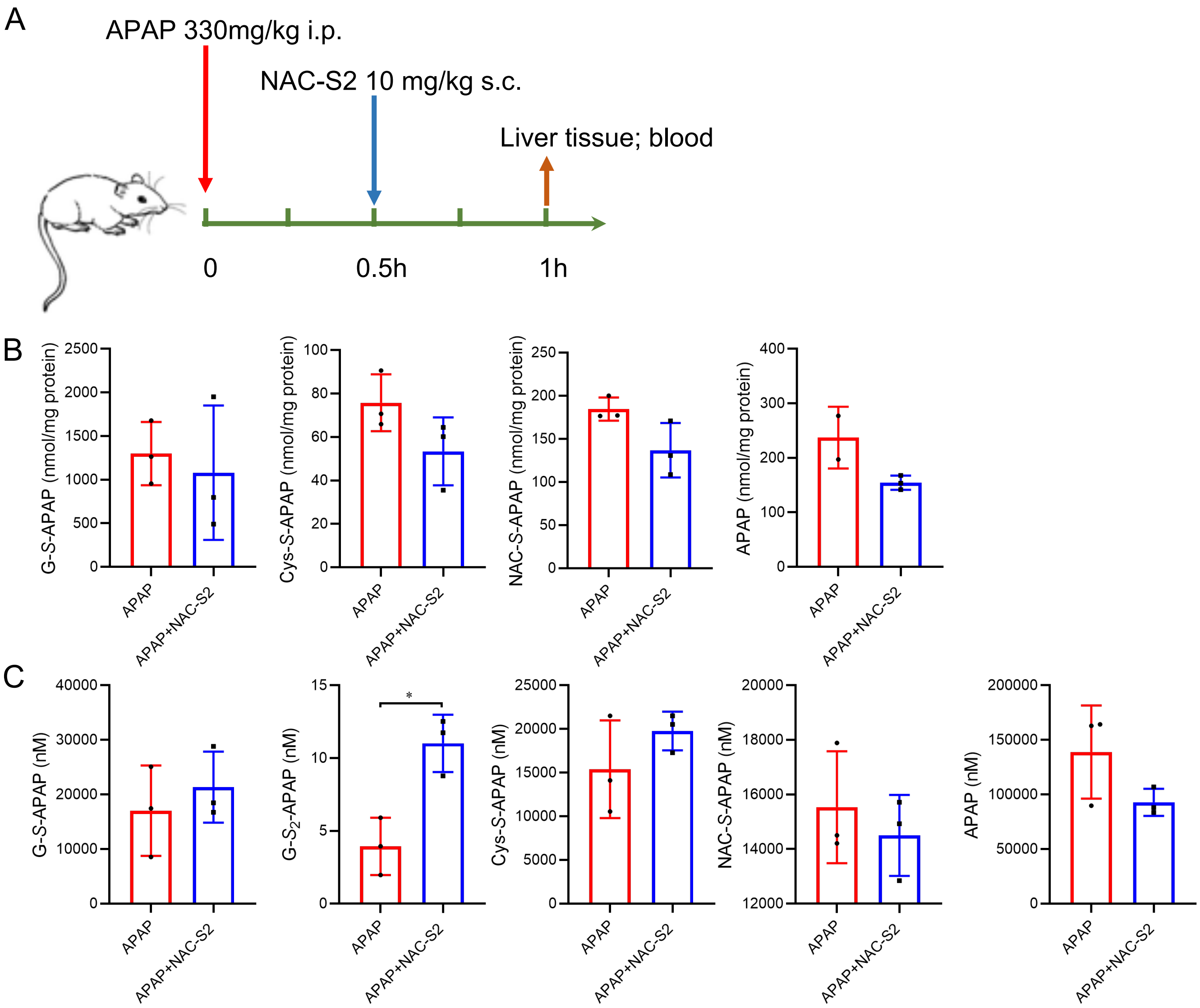

Supplementary Fig. S7. Effects of 10 mg/kg NAC-S2 on APAP adducts in mice. (A) The experimental protocol. Mice were treated with 10 mg/kg of NAC-S2 30 minutes after APAP injection, and liver tissues and blood were collected one hour after APAP injection. The levels of APAP-adducts, APAP-persulfide adducts, and APAP in (B) liver tissues and (C) blood were quantified by LC-MS/MS. Dates were expressed by means ± SD (n = 3). Statistical analysis was performed using Student's *t*-test. $^{*}P < 0.05$.

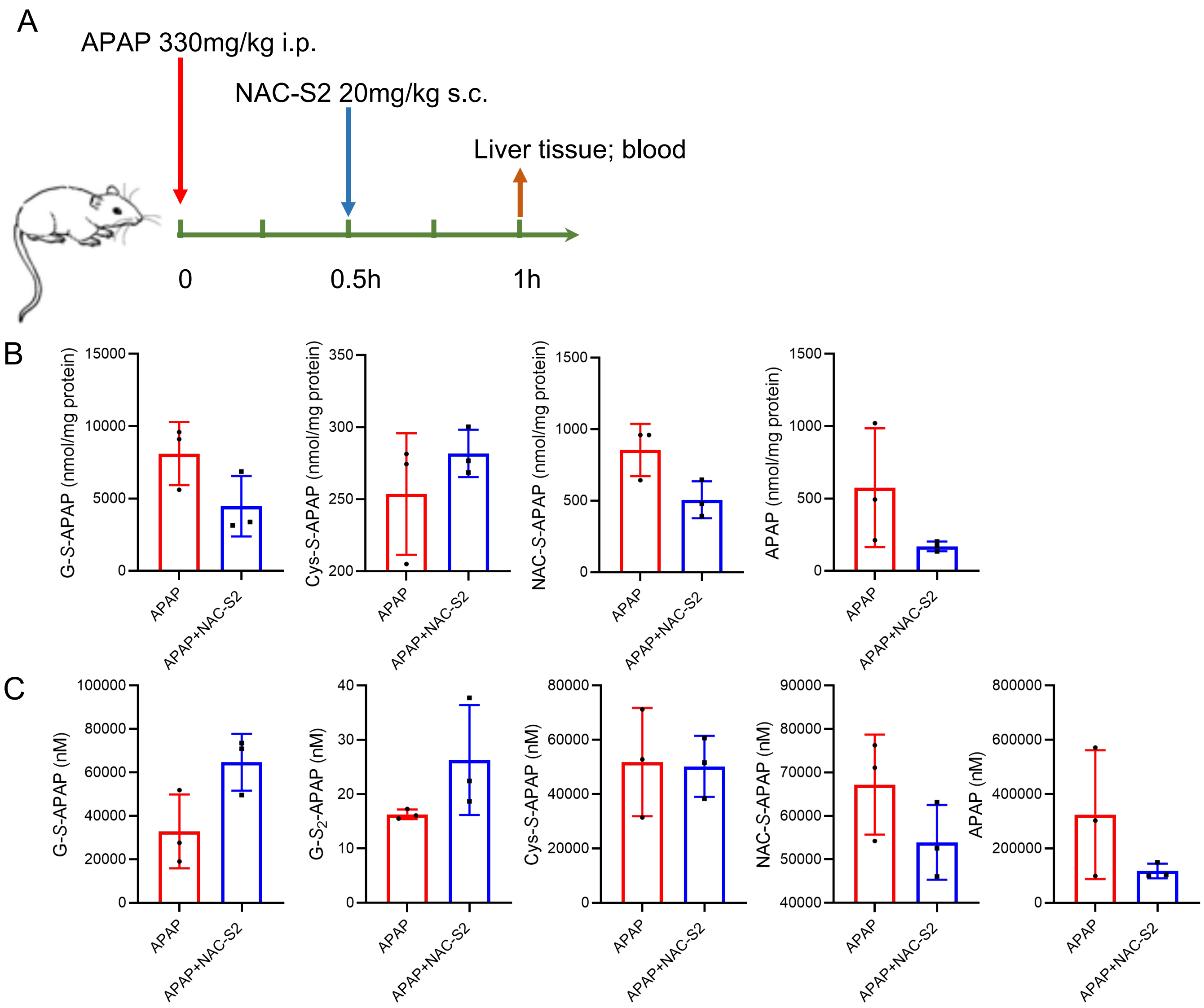

Supplementary Fig. S8. Effects of 20 mg/kg NAC-S2 on APAP adducts in mice. (A) The experimental protocol. Mice were treated with 20 mg/kg of NAC-S2 30 minutes after APAP injection, and liver tissues and blood were collected one hour after APAP injection. The levels of APAP-adducts, APAP-persulfide adducts, and APAP in (B) liver tissues and (C) blood were quantified by LC-MS/MS. Dates were expressed by means ± SD (n = 3).

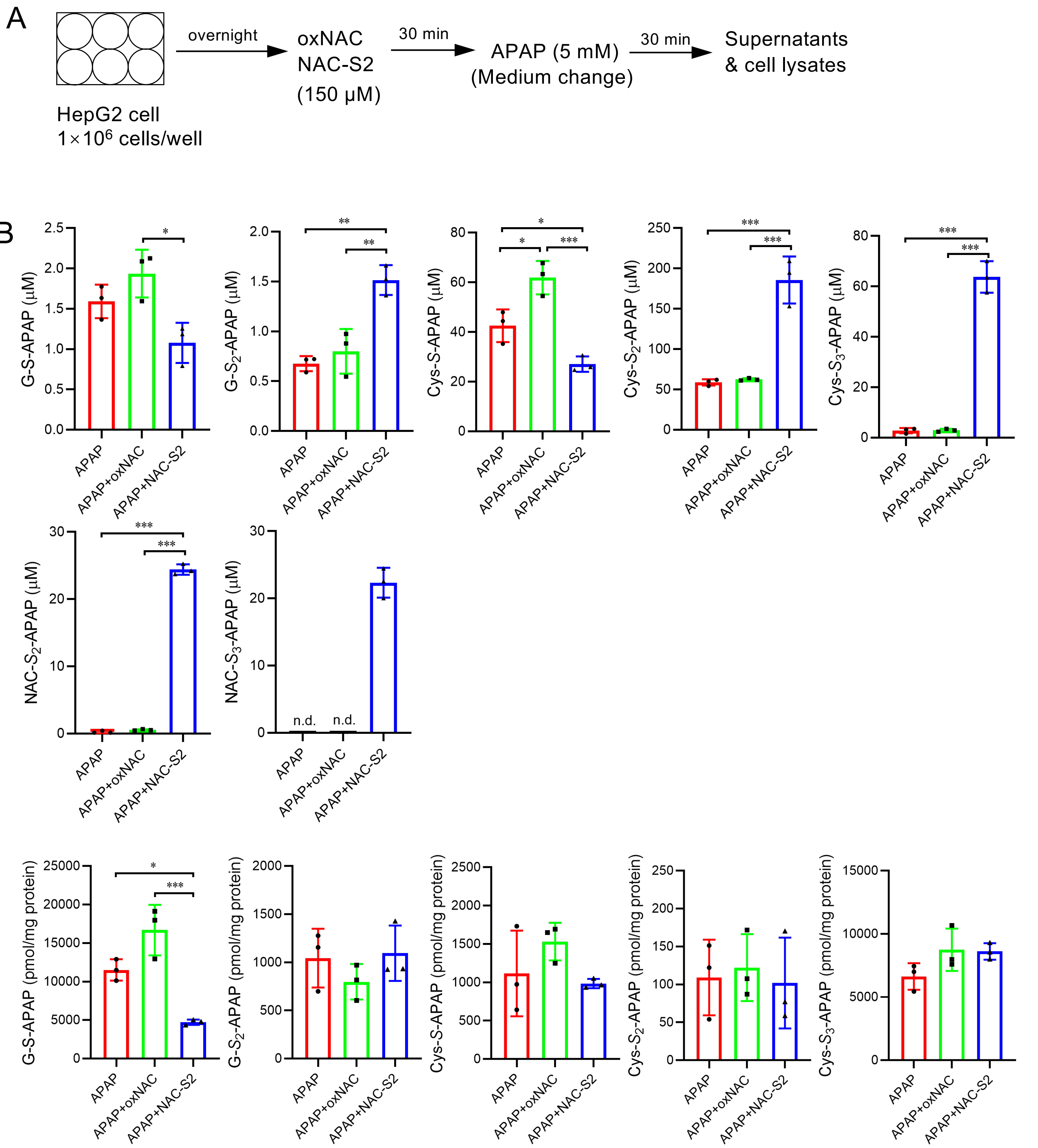

Supplementary Fig. S9. Formation of supersulfide–APAP conjugates in HepG2 cells and enhancement by NAC-S2 treatment. (A) Experimental protocol. HepG2 cells were treated with APAP for 30 min, after which APAP adducts in culture supernatants and cell lysates were analyzed separately. In addition, cells pretreated with oxNAC or NAC-S2 for 30 min prior to APAP exposure were also examined. Levels of APAP adducts in culture supernatants (B) and cell lysates (C). Data are expressed as mean ± SD (n = 3). n.d., not detected. Statistical analysis was performed using one-way ANOVA followed by Tukey's post hoc multiple comparisons test. $^{*}P < 0.05$, $^{**}P < 0.01$, $^{***}P < 0.001$.

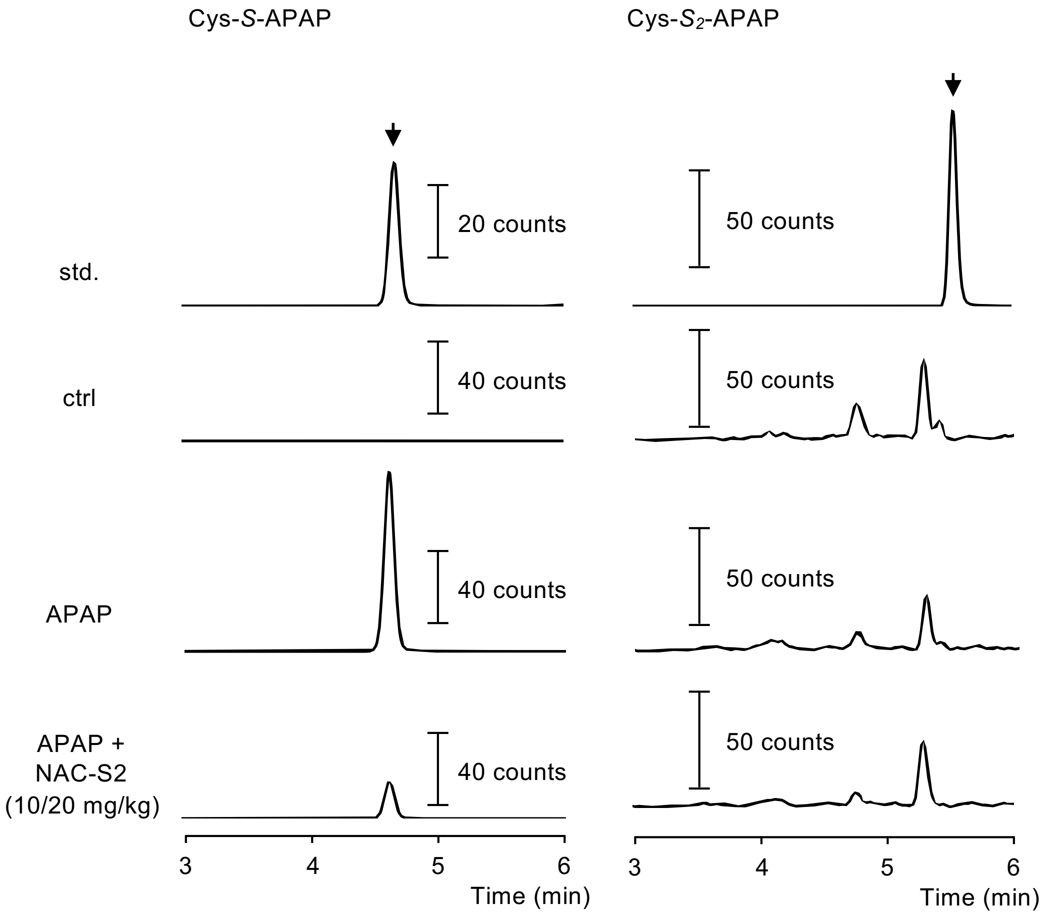

Supplementary Fig. S10. Representative mass chromatograms of protein-bound Cys-*S*-APAP and Cys-S_2-APAP adducts in liver tissues. Protein-bound APAP adducts were prepared as described in the main text. Mass chromatograms for Cys-*S*-APAP (left panels) and Cys-S_2-APAP (right panels) are shown. From top to bottom: standard (500 fmol/injection); control without APAP treatment; APAP administration (330 mg/kg); and APAP plus NAC-S2 administration (10 and 20 mg/kg).

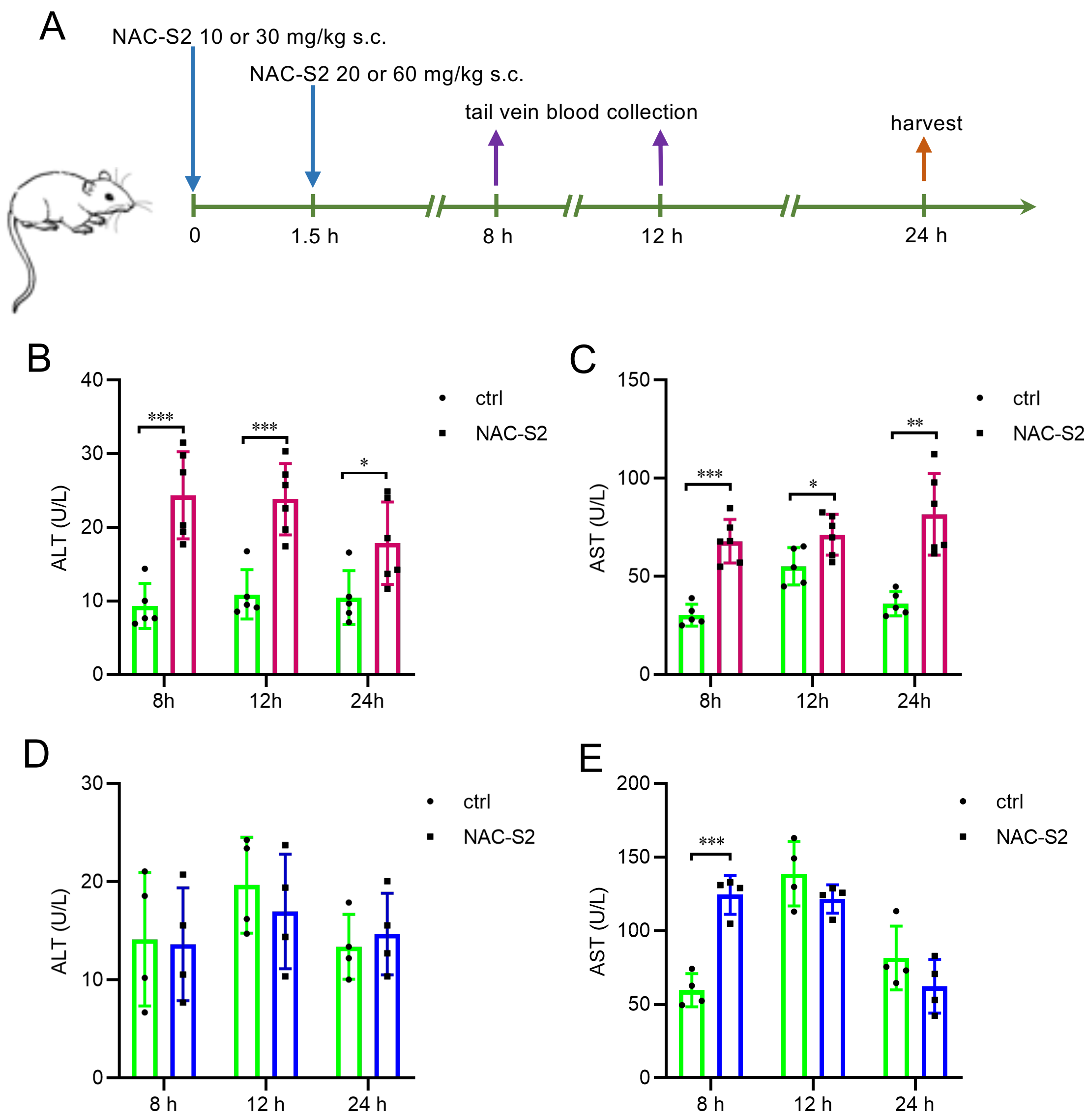

Supplementary Fig. S11. Safety profile of NAC-S2. (A) Experimental protocol. Mice were subcutaneously administered NAC-S2 at 0 h (10 or 30 mg/kg) and 1.5 h (20 or 60 mg/kg). Serum samples were collected at 8, 12, and 24 h after the first NAC-S2 injection. (B, C) Liver injury assessed by serum ALT and AST levels following NAC-S2 administration at 10 and 20 mg/kg. (D, E) Liver injury assessed by serum ALT and AST levels following NAC-S2 administration at 30 and 60 mg/kg. Data are expressed as mean ± SD (n ≥ 4). Statistical analysis was performed using Student's *t*-test. $^{*}P < 0.05$, $^{**}P < 0.01$, $^{***}P < 0.001$.

A

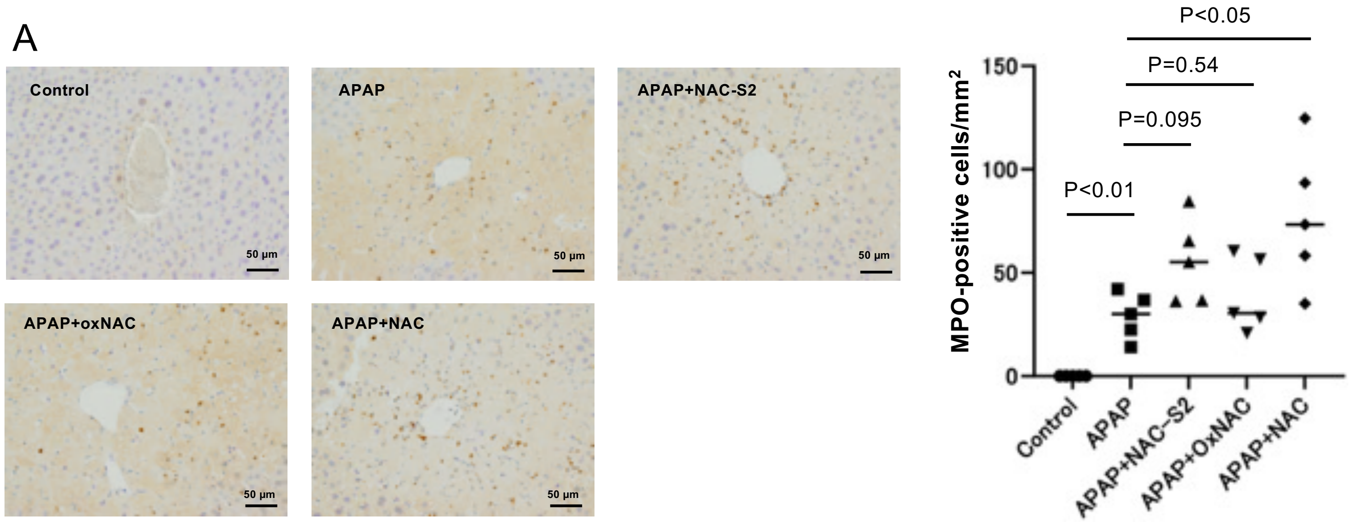

B

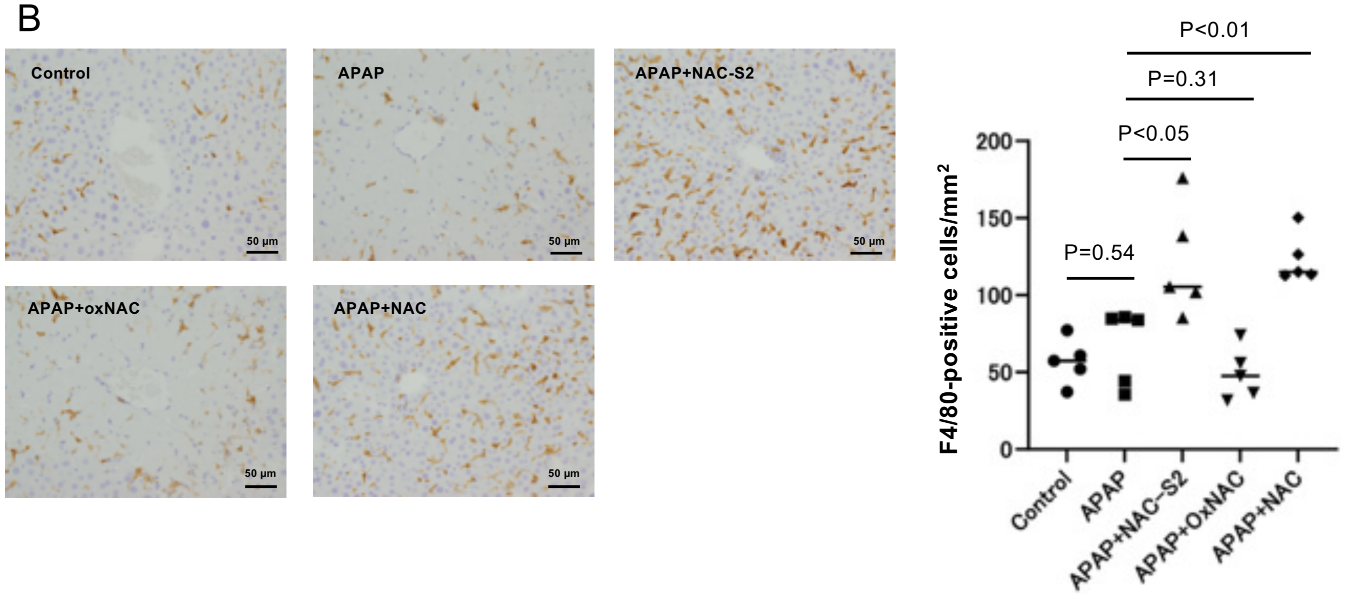

Supplementary Fig. S12. Immunohistochemical analysis of myeloperoxidase-positive cells and macrophages in the liver following APAP administration. Mice were intraperitoneally administered APAP (330 mg/kg), followed by subcutaneous injection of sulfur compounds (NAC-S2, oxNAC, or NAC) at 30 min and 2 h after APAP administration. Livers were harvested at 24 h post-APAP. Immunohistochemical staining for (A) MPO-positive cells and (B) macrophages was performed. Quantification of positively stained cells is also shown. Statistical analysis was performed using one-way ANOVA followed by Tukey's post hoc multiple comparisons test. Data were expressed as means ± SD ($n \geq 5$).

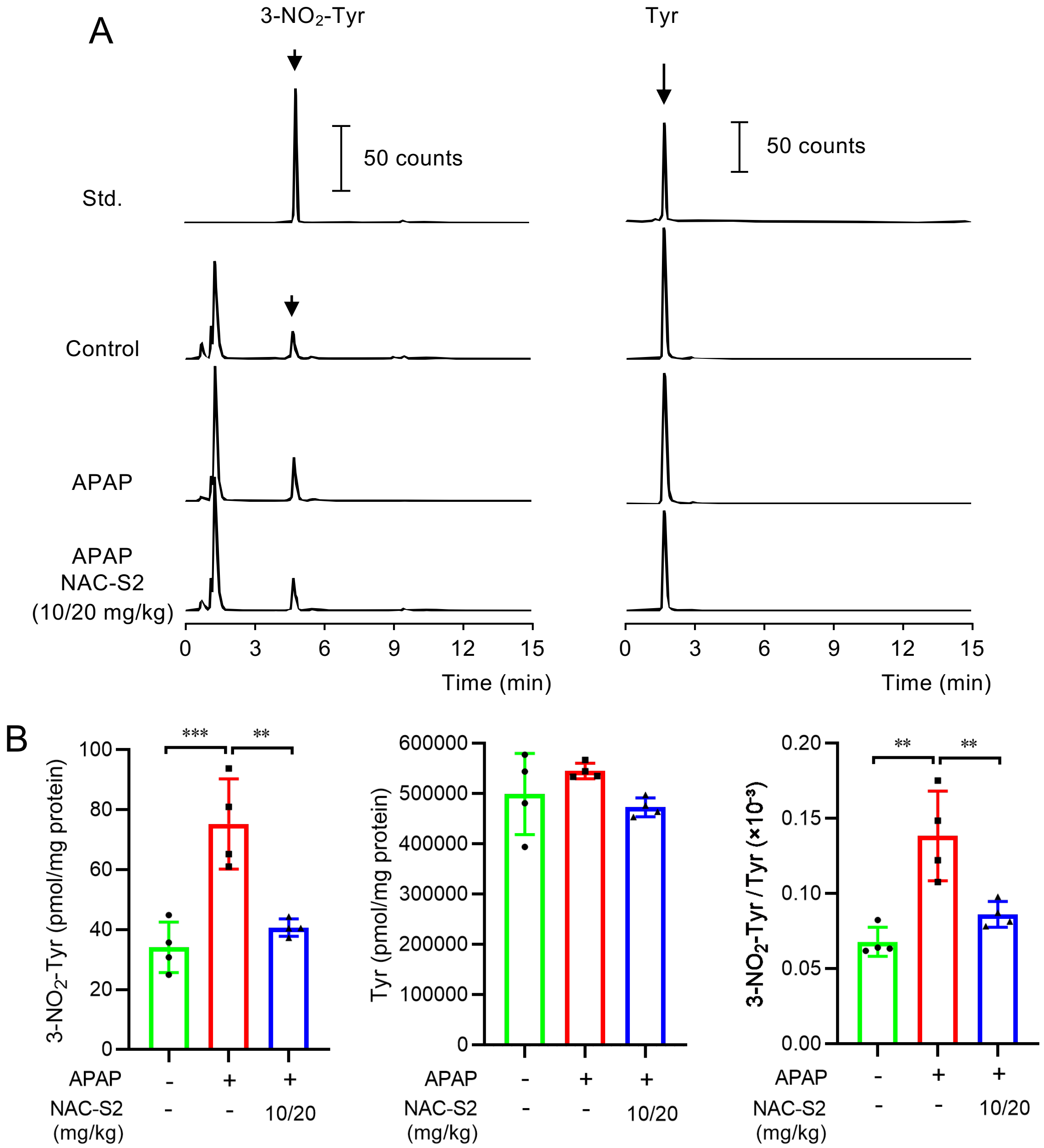

Supplementary Fig. S13. Tandem mass spectrometric determination of protein-bound tyrosine (Tyr) and 3-nitrotyrosine (3-NO_2-Tyr) in the liver following APAP administration. Mice were intraperitoneally administered APAP (330 mg/kg), followed by subcutaneous injections of NAC-S2 at 30 min (10 mg/kg) and 2 h (20 mg/kg) after APAP administration. Livers were harvested 24 h post-APAP. Mouse liver homogenates were subjected to ultrafiltration to remove free amino acids, followed by enzymatic digestion. Tyrosine and 3-nitrotyrosine formed during protein digestion were quantified by tandem mass spectrometry. (A) Representative mass chromatograms of authentic standards (0.1 pmol/injection), control tissue without APAP, APAP-treated mice, and APAP + NAC-S2–treated mice. (B) Quantitative data. Data are expressed as mean ± SD (n = 4). Statistical analysis was performed using one-way ANOVA followed by Tukey's post hoc multiple comparisons test. $^{*}P < 0.05$, $^{**}P < 0.01$, $^{***}P < 0.001$.

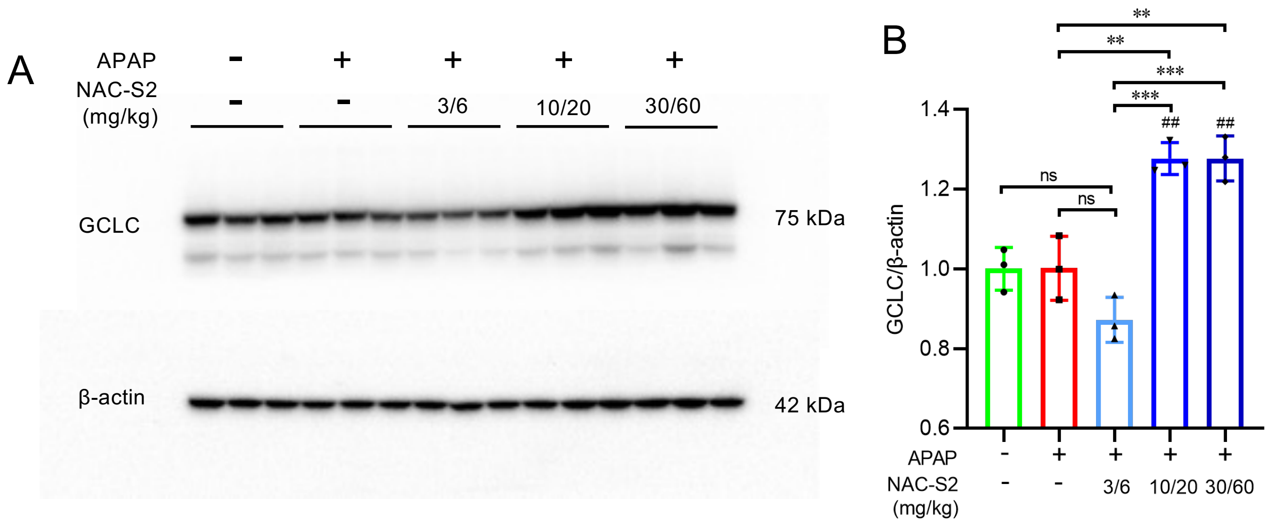

Supplementary Fig. S14. Effects of APAP overdose and NAC-S2 treatment on the expression of the glutamate–cysteine ligase catalytic subunit (GCLC) in liver tissue. Mice were intraperitoneally administered APAP (330 mg/kg), followed by subcutaneous injections of NAC-S2 at 30 min (3, 10, or 30 mg/kg) and 2 h (6, 20, or 60 mg/kg) after APAP administration. Livers were harvested 24 h post-APAP. (A) Western blot analysis of GCLC expression (Uncropped blots). (B) Quantification of Western blot band intensities. Relative protein levels were normalized to β-actin. Data are expressed as mean ± SD (n = 3). Statistical analysis was performed using one-way ANOVA followed by Tukey's post hoc multiple comparisons test. $^{*}P < 0.05$, $^{**}P < 0.01$, $^{***}P < 0.001$ vs. the indicated group; $^{\#}P < 0.05$, $^{\#\#}P < 0.01$, $^{\#\#\#}P < 0.001$ vs. the control group; ns, not significant.

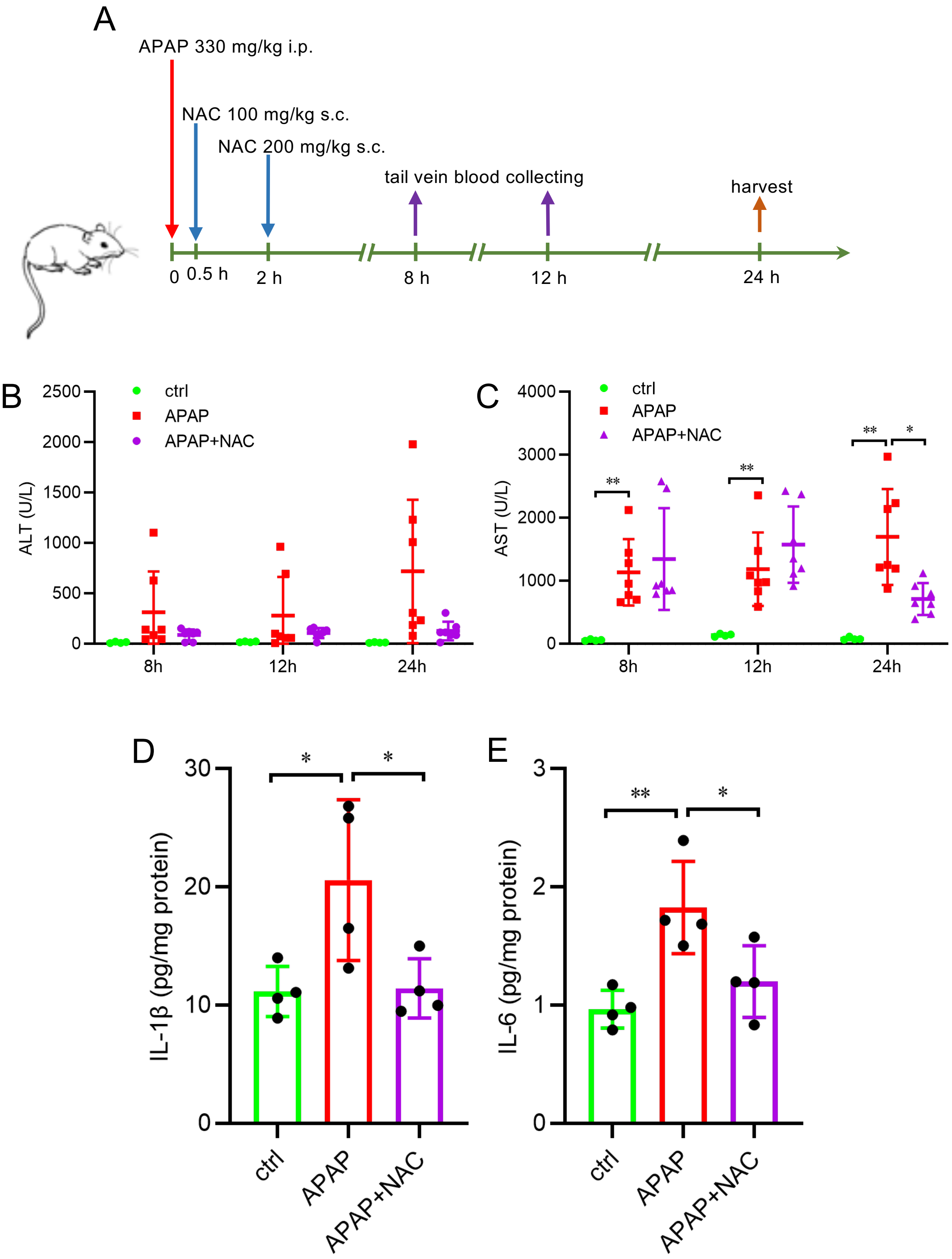

Supplementary Fig. S15. Effect of high-dose NAC on APAP-induced liver injury. (A) Experimental protocol. Mice were intraperitoneally administered APAP (330 mg/kg), followed by subcutaneous injections of NAC at 30 min (100 mg/kg) and 2 h (200 mg/kg) after APAP administration. Livers were harvested 24 h after APAP treatment. (B, C) Time course of serum ALT and AST levels. (D, E) Hepatic cytokine levels: IL-1β (D) and IL-6 (E). Data are expressed as means ± SD (n = 7 for B and C; n = 4 for D and E). Statistical analysis was performed using one-way ANOVA followed by Tukey's post hoc multiple comparisons test. $^{*}P < 0.05$, $^{**}P < 0.01$, $^{***}P < 0.001$.

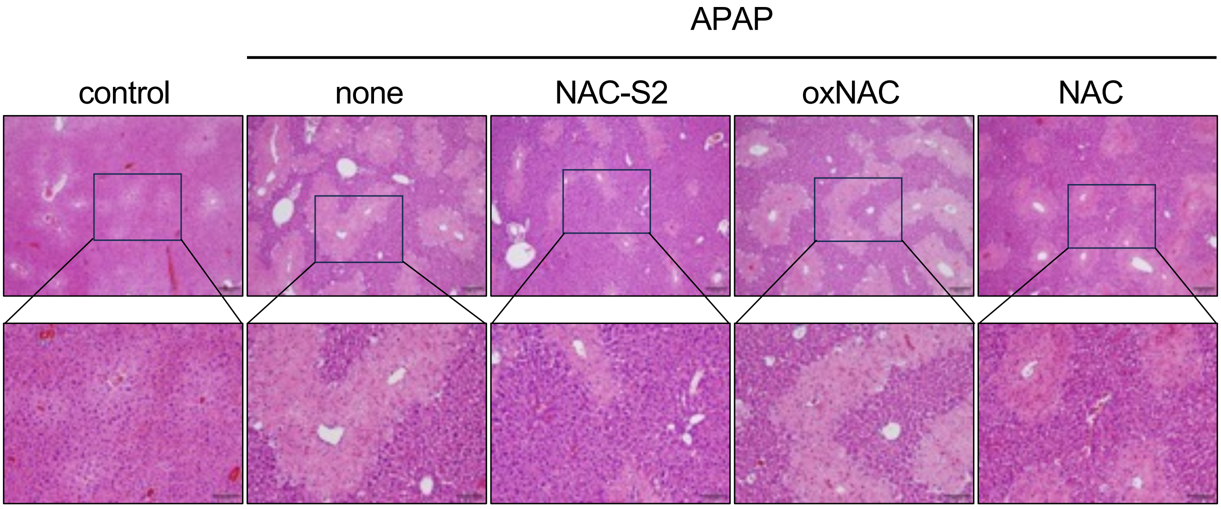

Supplementary Fig. S16. Effects of NAC-S2, oxNAC, and NAC on APAP-induced liver injury. Macroscopic appearance and H&E staining of liver tissues that used for quantification of necrotic area shown in Fig. 10D.

A

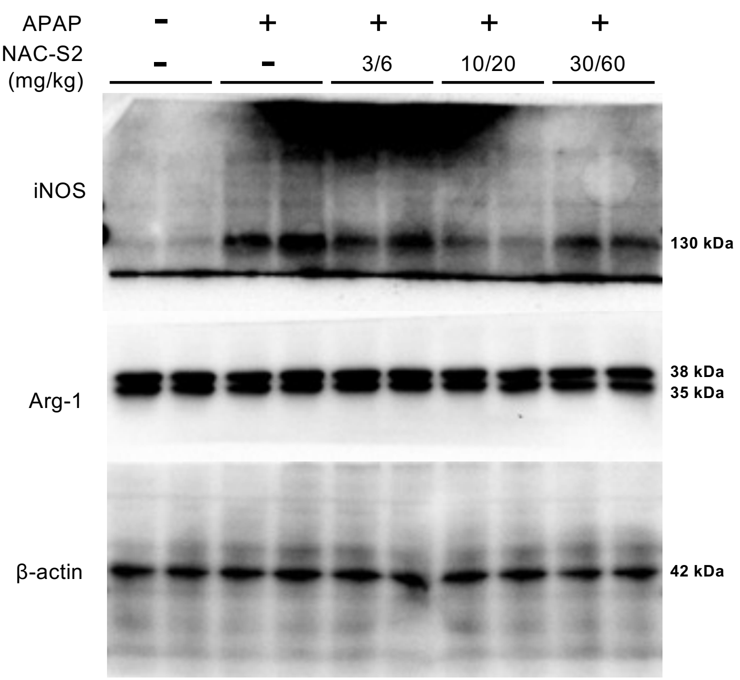

B

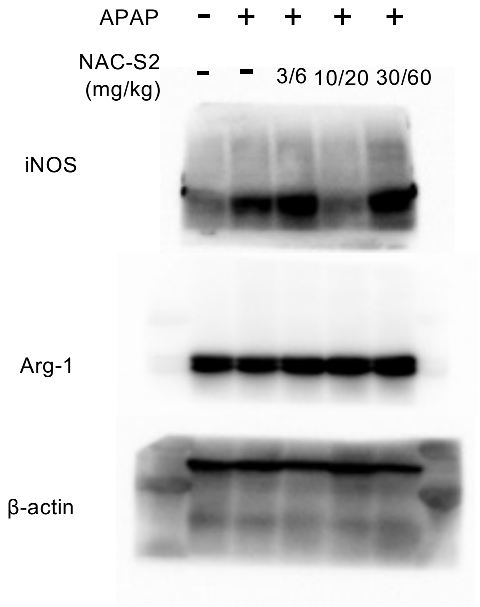

Supplementary Fig. S17. Full uncropped blot images from Fig. 9D. In (B), uncropped blot images for iNOS, Arg-1, and β-actin that were used for band intensity analysis (Fig. 9E, F) combined with (A).

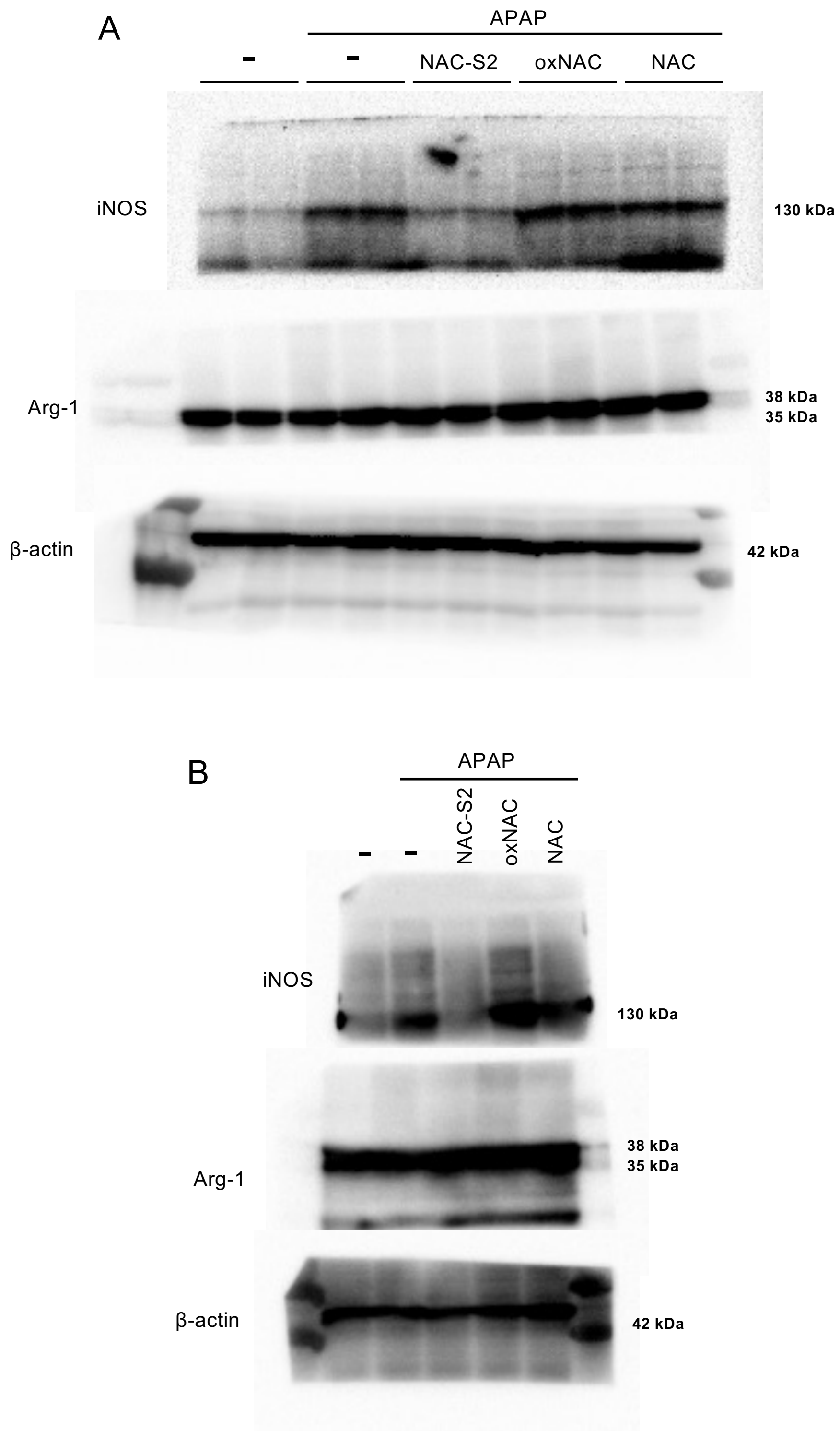

Supplementary Fig. S18. Full uncropped blot images from Fig. 10H. In (B), uncropped blot images for iNOS, Arg-1, and β-actin that were used for band intensity analysis (Fig. 8I, J) combined with (A).
